# Advancements and challenges in autonomous endovascular interventional robotics: A comprehensive review

**DOI:** 10.1016/j.isci.2025.114024

**Published:** 2025-11-14

**Authors:** Jingqian Sun, Ruichao Tang, Quan Zhang, Jiacheng Liu, Liming Gao, Weidong Yang, Xu Chen, Yichao Tang

**Affiliations:** 1 Shanghai Research Institute for Intelligent Autonomous Systems, Tongji University, Shanghai, China; 2 School of Mechanical Engineering, Tongji University, Shanghai, China; 3 Department of Cardiology, Shanghai East Hospital, School of Medicine Tongji University, Shanghai, China; 4 School of Aerospace Engineering and Applied Mechanics, Tongji University, Shanghai, China; 5 Shanghai Institute of Aircraft Mechanics and Control, Shanghai, China; 6 National Clinical Research Center for Interventional Medicine, Interventional Medicine Innovation Alliance, Shanghai, China; 7 Shanghai Innovation Institute, Shanghai, China

**Keywords:** robotics, applied sciences, engineering

## Abstract

Cardiovascular and cerebrovascular diseases remain major global health challenges. Minimally invasive endovascular interventions have become preferred treatments due to reduced trauma, pain, and recovery time. However, the advancement of endovascular interventional robotic systems (EIRSs) is constrained by limited imaging depth, heavy dependence on physician experience, and restricted device maneuverability. This review provides a comprehensive overview of the sensing, control, and execution modules that form the core of these EIRSs. It analyzes the fundamental challenges within each component and explores potential strategies to enhance system intelligence and autonomy. By integrating current progress with forward-looking insights, the review highlights pathways toward safer, more efficient, and greater autonomous robotic interventions. The ultimate aim is to advance the development of next-generation robotic platforms that can improve procedural precision and broaden patient access to high-quality vascular care.

## Graphical abstract

**Figure:**
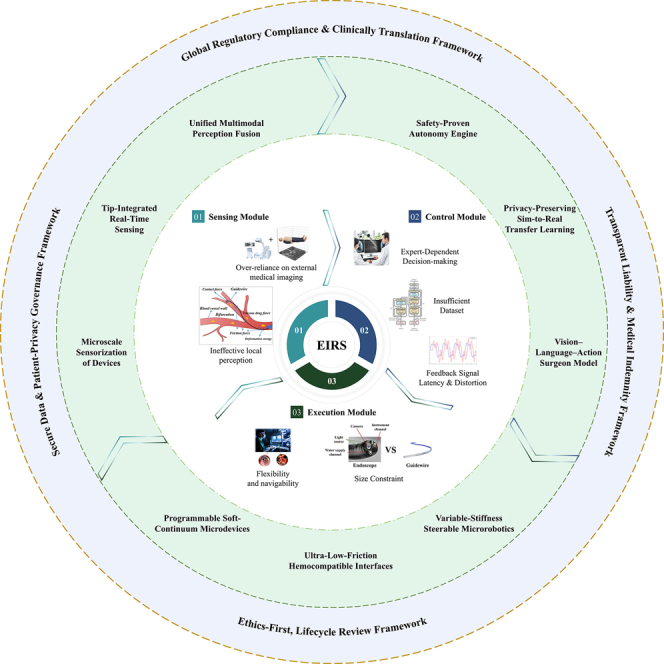

## Introduction

Cardiovascular and cerebrovascular diseases (CCVDs) are broadly defined as ischemic or hemorrhagic disorders of the heart, brain, and peripheral tissues caused by factors such as hyperlipidemia, increased blood viscosity, atherosclerosis, and hypertension. Owing to their high prevalence, disability rates, and mortality, CCVDs pose a serious global threat to health and have become a pressing public-health challenge.^1^^,^^2^ In 2019, CCVDs caused an estimated 17.8 million deaths worldwide, ranking first among all disease-related causes of death reported by The Lancet in 2020.^3^ CCVDs not only seriously affect individual health and quality of life but also bring heavy social and economic burdens.

Current treatment options for CCVDs fall into two broad categories: open surgical treatment and minimally invasive interventional surgery (MIIS). Open surgery provides direct access to the lesion area through open cranial or thoracic procedures. However, it is more invasive, entails longer recovery, and carries higher risks of complications, such as infection and bleeding, posing particular challenges for elderly, or frail patients.^4^

MIIS refers to a treatment method in which a few millimeter-sized incision is made in the aorta of the patient’s leg or arm with the assistance of medical imaging equipment, and a catheter and guidewire are pushed to the patient’s diseased location, and then therapeutic drugs or devices are delivered to these regions with the help of the catheter.^5^ MIIS has become an important breakthrough in modern medicine for the treatment of CCVDs. MIIS offers several advantages over traditional open surgery. It is less traumatic, reduces infections and pain, shortens recovery time, and minimizes scarring. The lower invasiveness also reduces the risk of postoperative complications like bleeding. These benefits make MIIS a preferred choice for treating CCVDs.^6^

However, the current paradigm of manual MIIS has several limitations. First, prolonged fluoroscopy exposes clinicians to cumulative ionizing radiation.^7^ Second, due to the complexity of the vascular structure, precise positioning of the guidewire or catheter often requires multiple attempts, increasing the risk of vascular injury. Third, the lead apron worn to protect the physician from radiation exposure tends to cause physician fatigue, which affects the stability and precision of manual manipulation during long procedures.^8^^,^^9^ Fourth, there is a long learning curve for skills in the use of MIIS techniques and devices.^10^ In addition, traditional interventional procedures rely heavily on catheters, guidewires, imaging systems, and other ancillary equipment. This reliance on advanced technology limits the accessibility and adoption of MIIS, particularly in resource-limited settings.

To overcome the drawbacks of the traditional manual MIIS paradigm, endovascular interventional robotic systems (EIRSs) have been introduced. In recent years, both academic and industrial research in this field has made significant progress. Several commercially available remote navigation systems have been developed for clinical applications, namely, the master-slave (teleoperated) interventional surgical robotic system.^11^^,^^12^^,^^13^^,^^14^ These systems allow interventionalists to remotely manipulate instruments such as catheters and guidewires from a shielded workstation, thereby eliminating the need to wear heavy lead aprons and significantly reducing radiation exposure.^15^ In addition, research teams are developing autonomous navigational EIRSs to further reduce the physical workload and operational demands on physicians, enabling them to focus on the accuracy, stability, and safety of the procedure.^16^^,^^17^^,^^18^^,^^19^^,^^20^^,^^21^^,^^22^^,^^23^^,^^24^

The evolution of EIRSs began in the early 2000s with the introduction of the Niobe system by Stereotaxis,^25^ the first robotic system to employ magnetic navigation for intravascular catheter manipulation. Hansen Medical introduced the Sensei X system, utilizing robotic arms to enhance surgical stability and precision. In 2012, Corindus Vascular Robotics’ CorPath 200 became the first FDA-approved robotic system for percutaneous coronary intervention. The CorPath GRX launched in 2016, incorporating automated motion and enhanced imaging capabilities. Robocath’s R-One system, CE-certified in Europe in 2019, featured guidewire locking and dual-retention mechanisms.^26^ Entering the 2020s, research has focused on developing next-generation robotic systems with biomimetic manipulation capabilities. These systems can autonomously navigate intravascular pathways, advance and retract devices, and even deploy stents.^27^ Such advancements have substantially improved the precision, safety, and efficiency of intravascular procedures, offering a promising alternative to conventional techniques. However, these systems are not yet compatible with all devices and may require intermittent manual intervention. Despite these limitations, their potential in endovascular interventional surgery remains substantial.

Although research on EIRSs has made notable progress, many key questions remain. For instance, why are the development of fully autonomous EIRSs necessary, and what technical limitations constrain autonomy? This review examines the development of fully autonomous robotic systems for endovascular procedures. It analyzes the workflow step by step, identifies key stages, and summarizes the core challenges currently faced. By surveying and analyzing relevant technologies, this paper aims to propose feasible solutions to these challenges and to outline future directions and potential opportunities for fully autonomous EIRSs.

## Challenges

**Figure 1:**
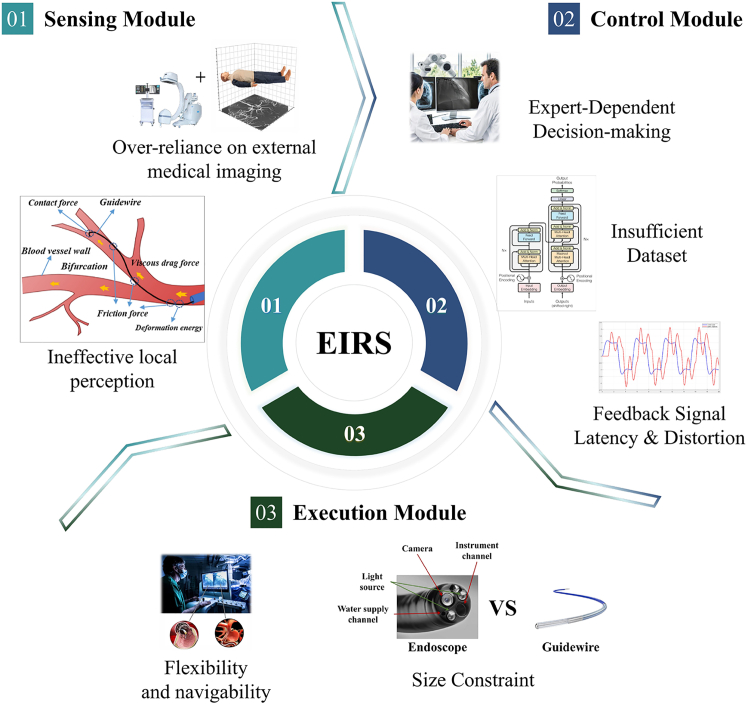
System overview of the EIRS and module-specific limitations The EIRS comprises: (1) sensing module, (2) control module, (3) execution module. (a) Sensing module: predominantly depends on extracorporeal imaging (e.g., fluoroscopy), with limited intraluminal interaction information (force/shape/contact) about the robot-environment interface. (b) Control module: most systems remain master-slave and expert-driven; attempts at autonomy face dataset scarcity, while feedback suffers from latency and distortion, limiting safe closed-loop performance. (c) Execution module: existing catheters/guidewires lack sufficient flexibility and steerability for small, tortuous vessels; miniaturization constrains sensor integration compared with larger endoscopic platforms. Figure reproduced with permission from: (1) sensing module—ineffective local perception^28^; (2) control module—insufficient dataset.^29^

An EIRS mainly contains the following three constituent modules: a sensing module, which performs multimodal data acquisition and preliminary environment understanding; a control module, which is further subdivided into decision-making layer (high-level surgical strategy and path planning) and low-level control layer (real-time motion and force regulation translating plans into control commands); and an execution module, which comprises the mechanical actuators and continuum robotic structures that physically realize the planned maneuvers (as illustrated in Figure 1). In traditional manual procedures, the physician is involved in every step of the process and plays a decisive role. To address the shortcomings of manual approaches, master-slave interventional robotic systems have emerged. However, this technology is a semi-autonomous system that realizes mechanical substitution only in the execution module. The physician still retains a significant role in the sensing and control modules. In order to further enhance system autonomy, the physician’s involvement in repetitive surgical tasks must be minimized, allowing them to focus on ensuring surgical accuracy and safety. This requires replacing manual functions in the sensing and control modules with autonomous approaches. For example, learning-based models can be employed to capture and replicate expert knowledge, enabling accurate interpretation of sensory data. Based on this analysis, the system can then generate safe and efficient control commands to guide the robotic platform in performing surgical tasks with greater precision and safety.

In an ideal autonomous EIRS, one of the most critical requirements is the robot’s ability to continuously and comprehensively perceive its vascular environment in real time. At the same time, it must accurately report its current pose information, including spatial position and angular orientation. This information is essential to ensure that the control module can generate safe and effective motion commands, avoiding harmful interactions between the robot and the delicate vascular structures. In traditional manual surgery, physicians rely on visual and tactile assessments to gather this information. Imaging data provide insights into the surrounding vascular structure and the robot’s location. However, relying solely on two-dimensional (2D) imaging may be insufficient for depth perception, making it difficult to assess spatial relationships accurately. To compensate, physicians use tactile feedback to enhance surgical safety. In contrast, EIRSs currently depend almost entirely on external medical imaging to perceive the surgical environment. This heavy reliance on visual data presents critical challenges to achieving the same level of safety that an experienced physician provides through combined visual and tactile feedback.

To better conceptualize these system-wide challenges and to map a clear trajectory toward higher levels of intelligence, it is crucial to characterize the autonomy of EIRSs in a structured manner. Inspired by existing autonomy frameworks in medical and surgical robotics,^30^^,^^31^^,^^32^^,^^33^ we propose a tailored autonomy grading scheme for EIRSs (summarized in Table 1). This scheme is closely aligned with the intrinsic architecture of sensing—control—execution and captures the progressive substitution of the physician’s role across the three modules, from mechanical execution to perceptive understanding and independent decision-making. By serving as a quantitative reference, the proposed grading framework (Table 1) not only facilitates an objective assessment of current technologies but also provides a roadmap to guide research priorities in each module. With this framework as a reference, the following sections discuss in detail the challenges that must be addressed within the sensing, control, and execution modules to enable EIRSs to advance through these autonomy levels.

**Table 1:** Proposed autonomy grading scheme for EIRS based on sensing-control-execution architecture

Level	Description	Sensing Module	Control module		Execution module
			Decision-making layer	Low-level control layer	
L0 – No Autonomy	The entire procedure is manually planned and executed; the robot merely reproduces human motions without independent perception or decision-making.	H	H	H	H
L1 – Assisted Execution	The surgeon retains full task control, while the robot provides motion scaling, tremor suppression, and safety boundaries to improve precision.	H/R	H	H/R	H/R
L2 – Task Autonomy	The system can autonomously perform limited subtasks (e.g., short-segment navigation) under human-defined goals; the surgeon supervises and authorizes each subtask while still engaging in parts of the operation.	H/R	H/R	R/H	R/H
L3 – Conditional Autonomy	The robot proposes and executes multi-step plans (e.g., full branch navigation) with minimal supervision; the human mainly oversees progress and intervenes only when necessary.	R/HS	R/HS	R	R
L4 – High Autonomy	The robot autonomously completes the entire endovascular procedure, including planning and real-time adaptation; the human remains only as a high-level safety supervisor.	R	R/HS	R	R
L5 – Full Autonomy	The robot independently performs full preoperative planning, intraoperative decision-making, and postoperative optimization without human intervention, achieving expert-level safety and efficacy.	R	R	R	R

The table defines six autonomy levels (L0—L5) for EIRS and uses refined human involvement codes: H = human dominant, HS = human supervision, R = robot dominant, H/R = human-dominated with robotic assistance, R/H = robot-dominated with limited human participation, and R/HS = robot-dominated with human supervision.

### Limitations of the EIRS sensing module: The “world map” problem

Currently, the commonly used sensing means in existing studies mainly include “world map sensing” and “machine-biology interface sensing”.

A common method of world map sensing in medical procedures involves imaging the vascular system using external medical imaging devices, with X-ray-based digital subtraction angiography (DSA) being the most commonly used. DSA can provide a “world map” for the interventional surgery, capturing the vascular anatomy, and tracking the position and movement of interventional tools. It gives a general map to guide the operation, helping physicians or artificial agents monitor both the interventional tools and the surrounding environment. However, in guidewire- and catheter-based interventions, using DSA requires the injection of contrast agents into the vessel to maintain image resolution and quality.^34^ Without angiography, autonomous identification or segmentation of vessels becomes difficult. Skilled physicians may be able to navigate a guidewire without using contrast agents when working in less critical areas, like non-tortuous vessels.^35^ Unfortunately, for EIRSs with inefficient tactile sensors, the lack of angiography leads to a loss of visual feedback, which in turn affects the safety of autonomous navigation, unless contrast agents are continuously injected. However, using high doses of contrast agents *in vivo* can result in severe side effects or even death.^36^ This reliance on angiography makes DSA impractical as the sole sensing device for autonomous EIRSs.

More importantly, DSA only provides 2D images, lacking depth information, making it challenging to capture the true spatial and temporal details of the vessels and interventional robots. As a result, it does not provide enough information for the decision-making submodule to make accurate navigation and surgical decisions. One potential solution is to use a preoperative model generated by computed tomography angiography (CTA),^37^ which adds depth information to 2D images, creating a three-dimensional (3D) “world map.” By registering real-time DSA images with this pre-obtained CTA map, it creates a 3D global positioning system (GPS) for the interventional robot, allowing for tracking the pose and trajectory of the robot.^38^ Nevertheless, registering CTA data with DSA imaging does not remove the need for contrast agents in autonomous navigation. It remains too risky to navigate the robot without continuous contrast imaging. In addition, preoperative CTA imaging cannot capture the dynamic nature of the circulatory system, particularly for vessels near the heart. These vessels continuously vibrate at unpredictable frequencies and amplitudes, and due to their soft, deformable nature, it becomes extremely difficult to register DSA images with CTA data accurately. Therefore, whether in 2D or 3D, relying on a global “world map” to guide the guidewire or catheter may be practical for skilled physicians, but it presents significant technical challenges when transferred to autonomous robotic interventions.

To overcome the aforementioned “world map” problem,^39^ techniques such as electromagnetic (EM) sensing and fiber Bragg grating (FBG) sensing have been integrated into interventional robotics systems. These sensors provide 3D pose/shape information during the movement of the interventional robot, thereby compensating for the limitations of image-based techniques. However, they only capture the shape-changing information of the interventional robot itself and do not directly reveal the surrounding vascular anatomy. Therefore, it is necessary to align this sensor-derived 3D information with the 3D vessel model obtained from preoperative CTA to achieve accurate positioning and tracking of the interventional robot; this, in turn, reintroduces registration errors.

To address the intrinsic limitations of GPS-like “world map” sensing systems, “machine-biology interface sensing” (referred to as “interface sensing” hereafter) is introduced to enhance the sensing capability at the machine-biology interface and to compensate for the limited information provided by “world map” sensing modules. These sensors are typically mounted on the robot’s body—for example, at the distal tip—to enable direct interaction with and monitoring of the *in vivo* environment. Force sensors deployed at the guidewire tip can monitor the collision force with the blood vessel wall.^40^ Alternatively, proximal force can be measured at the drive side. With the processing of guidewire conditions or outputs from force sensors directly assembled at the execution side, it can approximately find the relationship between proximal and distal forces.^41^^,^^42^

In interface sensing, there are problems such as poor real-time acquisition and imperfect decoupling of sensing signals. For the contact feedback (based on distal and proximal forces), whether the signal is transmitted to a human haptic device or fed into a control model, communication and actuation delays and transmission-induced distortion can occur. Furthermore, the proximal force contains information about the distal force; however, decoupling the distal force is difficult due to the combined effects of tip-wall contact, distributed friction force between the guidewire and the vessel, the viscous force of the blood, and potential deformation energy. This results in inaccurate distal-force estimation.^43^ For the autonomous endovascular robots that do not require human operation, insufficiently grounded sensing information also limits effective feedback for the decision-making submodule. Additionally, making force sensors compatible with small medical tools such as guidewires is challenging, as miniaturizing the sensor often compromises its accuracy, performance, and its ability to decouple normal, shear, and twisting forces.

In summary, the sensing module based on the “world map” exhibits several shortcomings that raise key questions: Which sensing modalities can efficiently compensate for missing dimensions in the “world map?” How can feedback be accurately decoupled in real time? Ultimately, how can multimodal information be fused effectively?

### Limitations of the EIRS control module

In traditional manual MIIS, interventional surgeons and patients face radiation exposure. Although the surgeon wears a lead apron during the procedure, there remains a risk of malignancy and cataracts. Cervical and lumbar spine injuries may also occur due to the bulk of lead aprons and the need to stand for prolonged periods.^44^ Therefore, EIRSs were developed to overcome these problems. However, most existing EIRSs follow a physician-operated paradigm (i.e., master-slave), in which the control module still depends entirely on the physician.^45^

In the traditional approach, the physician’s brain plays the role of the control module, while the hands serve as both the control and execute modules. When the hands perceive tactile feedback, the surgeon immediately integrates his clinical experience, knowledge of vascular anatomy, and interpretation of the 2D X-ray images. The surgeon then issues decision commands and controls the hands to execute them.^46^ In a master-slave system, however, the control and execution modules are realized by different subsystems. As a result, the surgeon must possess excellent device control ability in addition to the analytical abilities mentioned previously. Compared with the conventional method, the doctor still must sustain precise hand control for extended periods.^47^ Meanwhile, due to the “world map” problem and limitations of force sensing (see “Limitations of the EIRS Sensing Module” section), assessing the robot state becomes harder and, in turn, impairs device control. Therefore, the master-slave system not only fails to overcome the traditional dependence on physician experience but also increases training costs and time.^48^

Blood vessels are a dynamic environment, and the robot experiences contact and friction with the vessel wall during motion. Because the interventional robot has finite stiffness, such contact induces elastic deformation. Thus, control actions applied to the robot at the proximal end exhibit a nonlinear relationship with the response at the distal end. It is difficult to realize high-precision control of the apparatus by traditional control methods.^19^ Assuming a static vasculature under these conditions yields brittle state estimation and controller feedback. In practice, lightweight motion compensation is incorporated during mapping: (1) electrocardiogram/respiratory gating to “freeze” selected phases, but they require strict synchronization or breath-holding, limiting adaptability^49^^,^^50^; (2) dynamic coronary roadmapping aligning angiographic sequences with live fluoroscopy to reduce contrast and stabilize navigation, however, its performance may be degraded in patients with arrhythmia or catheter displacement^51^^,^^52^; (3) 2D/3D registration with non-rigid deformation for global compensation, though computational demands and robustness remain major challenges^53^^,^^54^; and (4) catheter/sensor-based motion modeling and prediction for closed-loop control, yet these approaches are hardware-dependent and sensitive to irregular rhythms.^55^^,^^56^^,^^57^ While each method has limitations, fusing these estimates yields a phase-consistent, time-resolved (3D + t) vascular map that may mitigate the “world map” problem and enhance the stability of robotic control.

Against this background, the development of autonomous systems is particularly important. With the advancement of artificial intelligence (AI), learning-based methods are increasingly used to solve various tasks.^29^^,^^58^^,^^59^^,^^60^ In recent years, several studies have attempted to achieve autonomous EIRSs using learning-based approaches, reporting promising results.^61^^,^^62^ However, learning-based methods still face challenges. High data-collection costs, limited interpretability, oversimplified training scenarios, and small datasets often yield models that are effective only for specific tasks and generalize poorly.^46^^,^^63^ In addition, some studies rely on virtual-environment simulation and then transfer the learned policy to the robot. Because simulations cannot fully reproduce real-world physics, performance in real environments is often unsatisfactory.^64^^,^^65^^,^^66^ Therefore, the simulation-to-real (sim2real) gap still needs to be addressed.

### Limitations of clinical surgical mechanisms in the EIRS execution module

Interventional surgeons encounter the “world map” problem when they operate medical tools under 2D X-ray fluoroscopy. The preferred devices for navigating instruments through lumens in this 2D world map are guidewires and catheters. In this navigation process, a guidewire-catheter system functions like a railway system, where the guidewire serves as the track and the catheter acts as the train carriage. Given lumen size constraints, a thin guidewire is the ideal choice for guiding the catheter due to its inherent advantages in size. The most common method to navigate a guidewire is to pre-shape its tip into a J-shape and apply insertion and rotation at the proximal end to control distal motion.^67^ During this process, the physician’s hands serve as both actuator and sensor.

Currently, most interventional robots are designed with the same sensing and operating principles as those used by physicians, whether they are master-slaved,^68^ automated, or autonomous. However, the inherent limitations of the “world map” problem pose technical challenges when transferring this guidewire-based navigation principle to robotic interventions.^69^ A key issue is that sensors mounted on the distal tip or body of the robot struggle to provide the same level of precise force feedback that a surgeon can feel, particularly those who are highly skilled and experienced (as discussed in the “limitations of the EIRS Sensing module” section).

This raises several open questions: What execution architecture is most suitable when “world map” constraints exist but accurate force feedback is unavailable? How can multimodal sensing improve the execution module? How can we design robots that explicitly address the “world map” problem?

To answer these questions, researchers are developing novel robotic structures and actuation mechanisms.^70^ New actuation strategies—such as fluidic, magnetic, and cable-driven mechanisms—are being explored to enhance flexibility and degree of freedom (DOF). Stiffness-variable continuum mechanisms are being developed to improve the robotic navigability, enabling them to adapt to stiffness- and size-changing lumens. Guidewire-free intervention concepts are also being investigated to replace guidewire-catheter systems during navigation. Nonetheless, the questions outlined previously remain challenging to answer.

## Current state of the art of ERIS

Based on the key challenges presented in chapter 2, this chapter presents a comprehensive literature review and current state analysis for each module of EIRSs. Through a systematic approach, it provides an in-depth examination and critical evaluation of existing technologies and methodologies. The analysis offers a structured summary of their advantages and limitations, as detailed in the following sections.

### Sensing module

**Figure 2:**
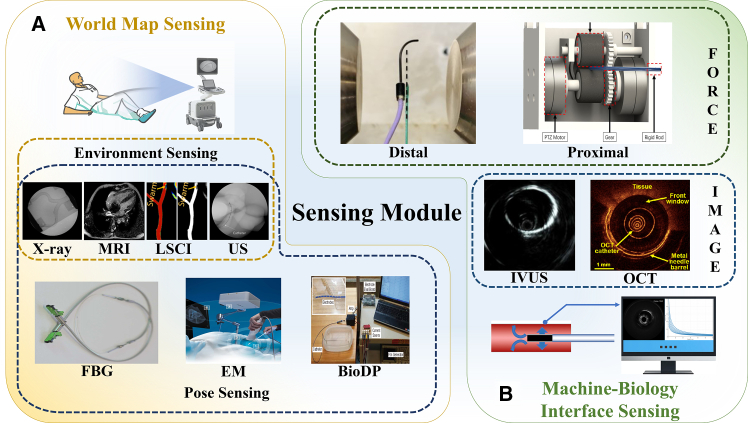
Classification of various sensors used in EIRSs Sensing modalities are grouped by function: (A) World map sensing supplies global information, which is categorized as environment sensing (X-ray,^71^ MRI,^72^ LSCI,^73^ and US^74^) and pose sensing (FBG, EM, and BioDP^75^). (B) Machine-Biology Interface Sensing captures local interaction, which is subdivided into force-based (distal sensor,^40^ proximal sensor^74^) and image-based (IVUS^76^ and OCT^77^). Abbreviations: MRI, magnetic resonance imaging; LSCI, laser speckle contrast imaging; US, ultrasound; FBG, fiber Bragg grating; EM, electromagnetic; BioDP, bio-dielectric property; IVUS, intravascular ultrasound; OCT, optical coherence tomography.

The sensing methods for EIRSs can be divided into world map sensing and interface sensing (as illustrated in Figure 2). For world map sensing, the primary limitation is the limited dimensionality of the sensing information. This manifests as difficulty in capturing the 3D intravascular environment in which the robot operates, leading to challenges in deriving navigational decisions and control strategies. For interface sensing, latency and signal distortion remain the main problems. This results in poor real-time transmission of force information and insufficient data for informed decision-making. Especially in master-slave systems, suboptimal human-robot interaction further exacerbates feedback distortion.

### Environmental sensing on the world map

#### X-ray fluoroscopy

The discovery of X-rays marked the beginning of the era of medical imaging. This breakthrough enabled physicians to visualize internal human structures noninvasively using X-ray imaging, thereby reducing the need for invasive diagnostic procedures. In the traditional paradigm of endovascular interventions, physicians rely on visual feedback from X-ray images to manipulate guidewires and catheters, steering them toward the target area during the procedure.^71^ However, fluoroscopic images provide only 2D representations and lack depth information, making accurate assessment of the surgical environment difficult. Furthermore, contrast agents are typically required to obtain clear fluoroscopic images, but their toxicity, together with X-ray radiation exposure, may harm patients.^78^

To compensate for the lack of depth information inherent in X-ray fluoroscopy, CTA is commonly employed to obtain preoperative 3D vascular data. CTA generates cross-sectional images of the patient’s body at varying depths through tomography, which can then be reconstructed into a 3D map using deep-learning models.^23^^,^^79^^,^^80^ This technique provides valuable 3D information, offering additional reference for surgical planning. However, it does not fully address the issues of X-ray radiation exposure, contrast-agent toxicity, or the absence of intraoperative 3D data. Moreover, because the vascular system is a dynamic environment that changes during the procedure, preoperative CTA data may become outdated or lose relevance as the intervention progresses. This leads to discrepancies between preoperative CTA data and real-time X-ray images during surgery.

#### Ultrasound sensing

With the introduction of medical ultrasound (US), physicians have gained an alternative imaging modality for visual tracking of catheters and guidewires.^81^^,^^82^^,^^83^ Compared with X-ray imaging, US imaging offers high soft tissue contrast and is relatively inexpensive to acquire. A key advantage of US imaging is that it does not involve harmful ionizing radiation. Moreover, the use of US imaging in catheter-based delivery experiments can enhance drug delivery efficiency and improve therapeutic outcomes.^84^^,^^85^ However, US imaging has limitations. It generally offers lower resolution and a smaller field of view compared with X-ray. Additionally, US imaging quality can be significantly affected by patient physiological motion (e.g., breathing and cardiac motion) and changes in body posture during interventional procedures.^86^ Beyond challenges associated with patient breathing and motion, ultrasound cannot penetrate the skull, thereby limiting its use in neurovascular interventions.^87^

#### Magnetic resonance imaging

To address many of the aforementioned challenges and improve surgical safety, magnetic resonance imaging (MRI) has been adopted as a preoperative alternative to X-ray fluoroscopy. MRI provides slice-by-slice 3D images, which are used for environmental analysis and modeling.^88^^,^^89^ MRI offers high-resolution images and a wider field of view, making it significantly more effective than US in terms of imaging quality. However, MRI-based interventional procedures face practical limitations. The strong magnetic field generated by the MRI system prohibits the presence of ferromagnetic materials within its operational area. As a result, all components of the EIRSs must be made from MRI-compatible materials, requiring specialized design and customization, which significantly increases costs. For certain endovascular interventions that require real-time continuous visualization, dynamic MRI presents an additional challenge. Whereas static acquisitions are readily achievable, such real-time imaging remains considerably more challenging due to limitations in temporal resolution (stemming from repetition time/echo time constraints and signal-to-noise ratio trade-offs), sequence and reconstruction latency, motion and susceptibility artifacts, and specific absorption rate (SAR)-related safety limits.^90^ Additionally, the MRI device surrounds the patient during operation, leaving a confined workspace that restricts procedural maneuverability and can render some procedures impractical. Therefore, MRI is primarily used for preoperative imaging, offering an advantage over preoperative CTA in terms of radiation-free imaging.^72^ Currently, some studies explore interventional robotic procedures within the continuous MRI environment, focusing on the deflection of magnetically controlled catheters in high-frequency or fringe-field magnetic environments.^91^^,^^92^ These efforts provide a foundation for interventional robotic research in MRI-guided operations.^93^

#### Laser speckle contrast imaging

Laser speckle contrast imaging (LSCI) for *in vivo* visualization is achieved by collecting and analyzing laser scattering information from blood cells. This technique enables the monitoring of robotic tip pose and blood flow dynamics.^73^^,^^82^ LSCI offers several advantages, including the absence of radiation exposure, compact, portable hardware, a wide field of view with high-resolution imaging, and noninvasive, real-time visualization. However, the technique has limitations. It often requires relative motion of the robotic body and temporary occlusion of vascular blood flow during imaging. Additionally, LSCI cannot penetrate deep or obstructed vascular regions and produces only 2D images.

**Figure 3:**
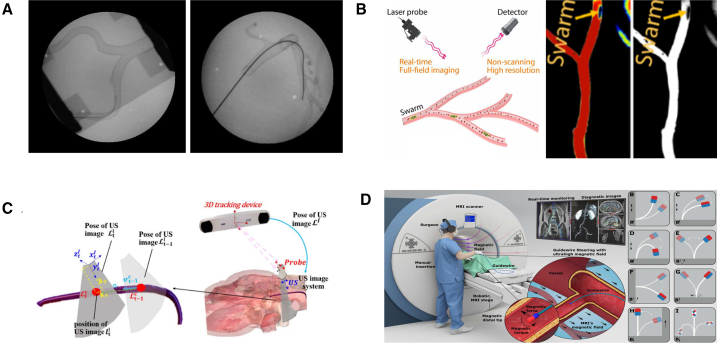
Comparative examples of environment imaging used for EIRS navigation Examples of environment sensing used in EIRSs, highlighting qualitative appearance and information content. (A) X-ray fluoroscopy^71^: wide field of view, device silhouettes for navigation. (B) LSCI^73^: laser-speckle-based perfusion/flow contrast. (C) US^83^: real-time soft-tissue/device visualization; tracked probe indicates imaging-plane pose. (D) MRI^93^: high soft-tissue contrast with intraprocedural guidance. Together, these outputs illustrate complementary contrast, resolution, and coverage.

The sensing methods listed previously (as shown in Figure 3) can provide information about the vascular structure, but they remain inadequate for acquiring detailed intraluminal information. To address this limitation, some studies have explored the use of conventional endoscopic techniques to obtain direct 3D environmental data. By flushing the endoscope lens with saline within a blood-filled artery, it is possible to acquire 2D images of the intravascular environment. However, low frame rates and bulky device sizes hinder the practical application of conventional endoscopes in the vasculature.^94^^,^^95^ Environmental sensing technologies enable the acquisition of both preoperative and intraoperative vascular data, providing critical support for subsequent surgical procedures by registering operative 2D data with preoperative 3D models. However, registering preoperative 3D models with 2D images acquired in a dynamic intraoperative environment can introduce alignment errors due to anatomical motion and viewpoint differences. Therefore, the development of mature and reliable intraoperative 3D sensing technologies has become a critical research focus.

#### Pose sensing on the world map

Real-time tracking of interventional robots is a critical aspect of autonomous EIRSs. Accurate real-time positioning of catheters and guidewires provides essential feedback for physicians or robotic actuators, guiding subsequent motion planning and execution. This capability is particularly vital in autonomous navigation scenarios, where precise positional data serves as the foundation for safe and effective operation. An apt analogy is GPS in autonomous vehicles: when the road ahead is blocked, GPS provides the precise location needed to adjust the route. Real-time tracking in endovascular interventions allows the autonomous EIRS to adapt its path based on the current position of the robot and the anatomical target. It is well known that catheter and guidewire insertion is a fundamental paradigm in interventional radiology and endovascular procedures. Therefore, achieving accurate tracking and integrating it into clinical workflows is increasingly essential for successful interventions.

Over the past decades, researchers have conducted extensive studies and made significant advancements in catheter and guidewire tracking. Currently, various tracking techniques have been developed, categorized primarily by sensor type into imaging-based, EM-based, FBG-based, and bio-dielectric property-based (BioDP) approaches. The following sections provide a detailed summary and analysis of each category.

#### Imaging-based pose sensing

**Figure 4:**
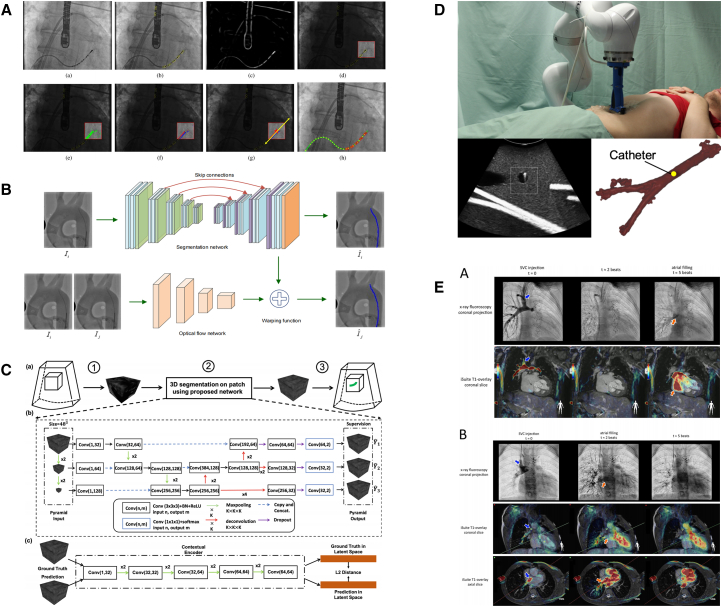
Imaging-based pose sensing for endovascular robotics: modalities and methods (A) X-ray, classical^97^; (B) X-ray, deep learning^106^; (C) US, deep learning^107^; (D) US, classical^102^; (E) MRI, classical.^105^ These examples highlight trade-offs (radiation, artifacts, data demands) and the alignment with world-map environment sensing.

In traditional surgical settings, surgeons primarily rely on imaging guidance to determine the position of the guidewire and catheter relative to the patient’s internal anatomy. Accurate recognition and extraction of the catheter and guidewire’s pose parameters are critical for providing precise navigational feedback to the EIRS during interventional procedures. These image-based pose-sensing techniques align closely with those used in “environmental sensing on the world map”. The use of image-based technologies in EIRSs includes X-ray fluoroscopy,^96^^,^^97^^,^^98^^,^^99^^,^^100^ US,^82^^,^^101^^,^^102^ and MRI^103^^,^^104^^,^^105^ (as illustrated in Figure 4). Among these, X-ray fluoroscopy was the earliest technique adopted. US and MRI, while offering distinct advantages and limitations compared with X-ray, are discussed in more detail in section “environmental sensing on the world map.”

With the advancement of deep learning-based medical image segmentation techniques, many researchers have explored the use of deep learning models for catheter and guidewire detection and tracking.^106^^,^^107^^,^^108^^,^^109^^,^^110^^,^^111^ However, these learning-based approaches are highly reliant on large amounts of labeled data, the acquisition of which is both labor-intensive and time-consuming. Furthermore, due to the nature of X-ray imaging, X-ray-based tracking methods inevitably expose both patients and staff to ionizing radiation. As a result, minimizing radiation exposure has become a key objective in the development of these technologies. In addition, tissue deformations due to the forces exerted on the US probe by the sonographer to ensure direct contact with the body surface can significantly impact US imaging results. Moreover, acoustic artifacts remain a major challenge for catheter and guidewire tracking based on US imaging.^112^

These image-based methods provide only 2D image information during procedures, resulting in a lack of depth information. To address this limitation and offer more intuitive 3D positional information, some researchers have attempted to register intraoperative 2D images with preoperative 3D data obtained through CTA. This approach aims to more accurately estimate the position of the device tip.^83^^,^^102^^,^^113^ While registration techniques have demonstrated promising performance in static environments, they face notable challenges in dynamic clinical settings. The time gap between the acquisition of preoperative 3D CTA data and intraoperative real-time 2D imaging can introduce registration errors. Additionally, in endovascular interventions, significant tissue deformation caused by vascular motion—particularly due to cardiac activity—further compromises the accuracy of the registration process.

#### EM-based pose sensing

**Figure 5:**
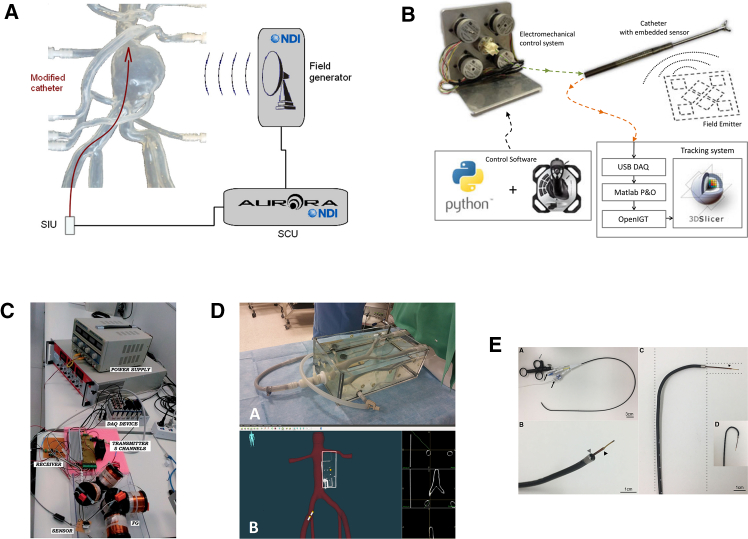
EM-based pose sensing for EIRSs: system architecture (MFG, EM sensor, SIU, and SCU) and representative implementations from commercial platforms and custom coil arrays used for real-time 3D tip localization Figure reproduced with permission from: (A)^114^; (B)^115^; (C)^116^; (D)^117^; (E).^118^

To overcome the limitations of image-guided robot tracking methods, which can only provide 2D information, some researchers have explored the use of sensors capable of directly providing 3D feedback. Among these, EM localization sensors are a leading technology frequently reported in recent studies (as shown in Figure 5). An EM localization system generally consists of four key components: a magnetic field generator (MFG), an EM sensor, a sensor interface unit (SIU), and a system control unit (SCU). When the EM sensor moves within the magnetic field generated by the MFG, the SIU collects real-time position and orientation data and transmits it to the SCU, which visualizes the 3D data on the host computer.

Some scholars have conducted vascular-intervention experiments to evaluate the performance and accuracy of EM localization systems in various endovascular scenarios.^114^^,^^117^^,^^118^^,^^119^ In addition, several studies have also explored the development of custom-built EM field generators, composed of EM coils, to enhance system sensitivity. By tuning coil arrangement to adapt to different application scenarios and catheter and guidewire sizes, researchers aim to optimize system performance.^115^^,^^116^ EM tracking technology is extensively researched in academia and is also available as mature commercial products. Well-established companies such as Northern Digital Inc-NDI (Waterloo, Ontario, Canada), Philips (Andover, Massachusetts, USA), and Siemens Healthineers (Malvern, Pennsylvania, USA) offer EM tracking solutions.^112^ As a result, researchers have convenient access to a wide range of EM sensors in diverse shapes and sizes. These sensors can be integrated into device tips to facilitate the development of EM-based catheter and guidewire tracking algorithms.

Although EM sensors can directly provide the 3D position and orientation of the catheter and guidewire tip, their application is still limited by certain drawbacks. First, due to their EM nature, EM tracking systems are susceptible to interference from nearby imaging devices, which may distort measurements. Second, the presence of ferromagnetic materials and equipment in the surgical environment can introduce further interference, degrading tracking performance. Third, the EM tracking system itself may introduce internal measurement errors when determining the EM sensor’s position relative to its coordinate system. Finally, the operational space of the interventional robot is limited to the spatial boundaries of the magnetic field generated by the MFG. If the surgical working area is extended by moving the MFG, discrepancies may arise in the positional data returned by the EM sensors, because these readings are not inherently tied to a consistent global coordinate system. This lack of a unified reference frame introduces additional registration errors.

#### FBG-based pose sensing

**Figure 6:**
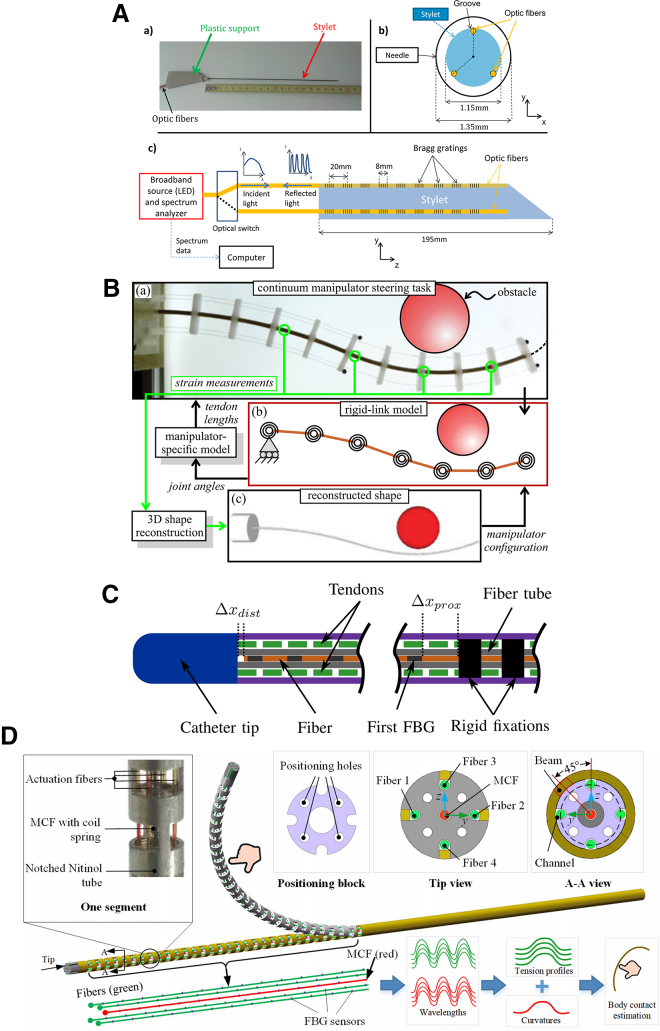
Demonstration of intervention robot positioning using FBG technology Representative implementations of FBG-based pose sensing for catheter/guidewire robots. Panels show architectures embedding distributed FBGs and reading them via a fiber-optic demodulator. Current studies emphasize optimizing fiber count and spatial routing and per-fiber FBG configuration/quantity to improve 3D shape reconstruction and tip localization. Figure reproduced with permission from: (A)^120^; (B)^121^; (C)^122^; (D).^123^

FBG-based sensing represents an emerging technology recently introduced into interventional applications (as illustrated in Figure 6). At its core, this technology involves an optical fiber embedded with FBG sensors, which are used to estimate changes in the fiber’s 3D shape. The working principle relies on detecting shifts in the specific wavelengths of light reflected by each FBG sensor. These wavelength shifts correspond to variations in strain along the optical fiber, which can be used to infer local deformations. By integrating the deformation data obtained from multiple FBG sensors distributed along the optical fiber, the overall shape of the entire fiber—and thus the interventional instrument—can be reconstructed. Currently, FBG-based studies in endovascular interventions primarily focus on optimizing the number and spatial arrangement of optical fibers embedded in the interventional robot, as well as the configuration and quantity of FBGs along each fiber.^120^^,^^121^^,^^122^^,^^123^^,^^124^

Compared with the previously mentioned techniques, FBG-based catheter and guidewire tracking offers several advantages, such as a small size, greater flexibility, immunity to EM interference, and ease of integration with other interventional robotic instruments.^125^ However, this method still has several limitations. The FBG-based approach requires the additional integration of a fiber optic demodulator into the interventional robotic system, which increases both system complexity and cost. Moreover, effective implementation of FBG-based tracking necessitates precise sensor registration and calibration, further complicating the setup and introducing additional technical challenges.

#### BioDP-based pose sensing

**Figure 7:**
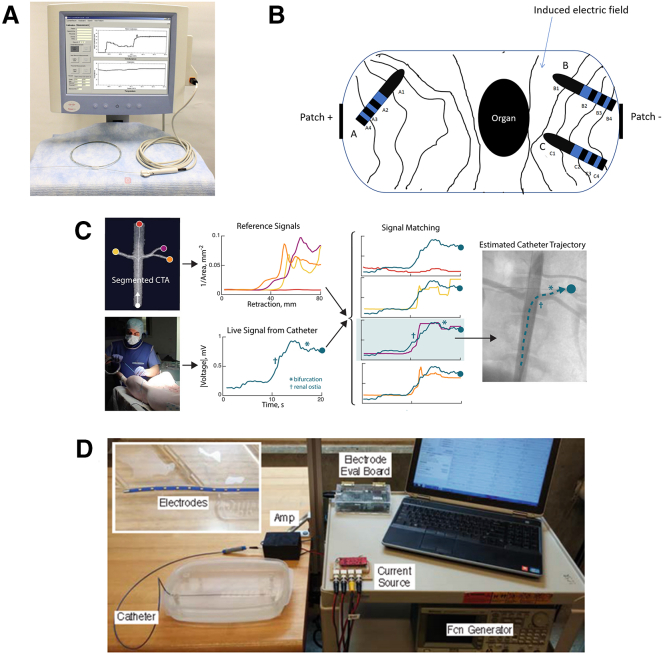
Representative implementations of BioDP-based pose sensing in EIRSs are shown (A),^126^ (C),^127^ and (D)^75^ tip electrodes generating weak fields with impedance mapped to preoperative X-ray/MRI models; (B)^128^ body-surface electrodes generating fields while a dielectric catheter senses voltage changes.

In addition to the conventional catheter and guidewire tracking methods discussed previously, some researchers have proposed utilizing biological dielectric properties to achieve real-time position tracking of interventional robots (as shown in Figure 7). This technique is based on the principle that biological tissues and organs exhibit specific dielectric responses when subjected to an electric field generated by electrodes. These responses lead to measurable changes in voltage or current signals, which can be captured by the system and converted into distance information, thereby enabling accurate catheter localization.

Some studies have integrated electrodes at the catheter tip to generate weak electric fields, allowing impedance signals from surrounding tissues to be detected. These signals are then mapped to preoperative impedance models derived from X-ray or MRI data to determine the position of the catheter tip.^75^^,^^126^^,^^127^ Additional research has involved attaching electrode sheets to the patient’s body surface to generate an electric field, with a dielectric catheter used to detect resulting voltage changes. The detected signals are then converted into distance data, enabling real-time tracking of the catheter tip’s position.^128^

**Figure 8:**
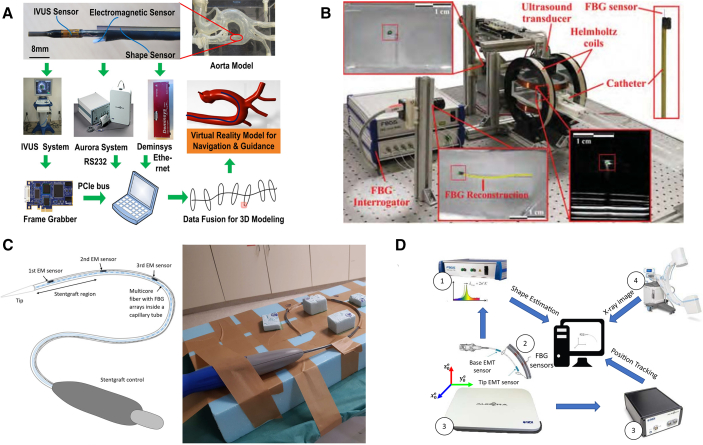
Representative implementations of intervention robot positioning using a hybrid of multiple sensors Figure reproduced with permission from: (A)^129^; (B)^130^; (C)^131^; (D).^132^

In addition to the aforementioned categories, some researchers have explored hybrid approaches that combine multiple sensing technologies to enhance tracking accuracy and robustness (as illustrated in Figure 8). The combination strategies investigated to date include: integrating EM and FBG sensors,^131^ combining US and FBG,^130^ using a tri-modal setup combining EM, FBG, and IVUS,^76^^,^^129^ and integrating FBG and EM with X-ray imaging.^132^

Among all the tracking techniques analyzed thus far, X-ray-based and US-based methods can only provide 2D intraoperative information. Moreover, X-ray-based imaging involves ionizing radiation, which may cause irreversible damage. In contrast, MRI-based systems can indirectly achieve 3D visualization by offering three orthogonal 2D views. However, they impose strict requirements on the experimental environment and equipment due to the system’s strong magnetic fields. Similarly, EM-based tracking systems face comparable challenges related to EM interference. Regarding FBG-based and BioDP-based methods, they are capable of directly capturing the 3D shape deformation of the interventional robot itself. However, they cannot accurately reflect the structure of the surrounding vascular anatomy unless registered with a preoperative 3D model.

### Interface sensing

#### Proximal force sensing

**Figure 9:**
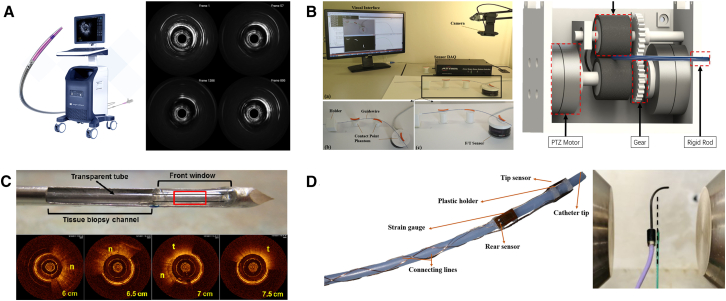
Interface sensing modalities in EIRSs with example outputs: intravascular imaging and distal/proximal force sensing Representative interface-sensing results in EIRSs. (A and C) Image-based sensing: IVUS/OCT visualize vessel wall, lumen, and device-wall apposition to guide interaction. (B and D) Force-based sensing: proximal estimates interaction via drive-train signals; distal measures contact forces at the tip. Together, these cues support safe manipulation and local feedback. Figure reproduced with permission from: B, left,^133^ right^74^; C^77^; D, left,^134^ right.^40^

Force sensing in interventional robotics is typically classified into proximal sensing and distal sensing, based on the location of the sensor (see Figures 9B and 9D). The demand for proximal force sensing initially arose to replicate the tactile feedback that clinicians receive during manual manipulation. Proximal force sensing is commonly achieved through dedicated force sensors or by calculating force from motor current feedback.^35^^,^^41^^,^^135^ However, traditional force sensors often introduce additional damping, distorting the sensed data. Similarly, motor current-based estimation requires high precision and stability from the actuators, imposing strict demands on system design and control robustness.^136^^,^^137^ Another indirect approach involves estimating proximal force by tracking displacement or deformation via visual methods.^133^^,^^138^ Although non-contact visual sensors eliminate mechanical interference, their accuracy is dependent on theoretical modeling, which can lack robustness under dynamic or variable conditions.

Recent studies have demonstrated the potential of deep learning models for estimating proximal forces with improved accuracy. These models combine distal deformation data with proximal force and torque measurements to infer the force distribution more precisely.^139^^,^^140^ However, a systematic standard for interpreting proximal force data are still lacking, making it challenging to decouple the proximal force signals accurately. For master-slave robotic systems, another challenge beyond acquiring sensing data are enabling the operator to perceive the same level of force feedback from the slave side. This is primarily addressed through force feedback systems on the master robot.

Traditional master robot interaction systems adopt rigid rocker-based control mechanisms, which provide visual feedback but lack tactile feedback. While this visual-only approach protects the operator, it significantly diminishes intuitive perception. To address this, various force feedback systems have been proposed that simulate the proximal force during the actual surgical conditions. These systems decompose motion into two modules based on the rotation and push-pull actions of the catheter or guidewire during surgery. The catheter push-pull module of the active robot typically employs damping-generating devices for sensing feedback, including motor feedback force transmission,^74^^,^^141^^,^^142^ magneto-fluidic force feedback,^143^^,^^144^^,^^145^ and friction feedback force transmission.^146^ Meanwhile, most catheter rotational feedback modules in active robots are driven by motor feedback. Recent work has also explored magnetic actuation-based force feedback, where Feng et al. designed a proximal haptic interface that reproduces slave-side interaction forces on the master side via magnetic actuation, thereby enhancing operational transparency.^147^ Despite these efforts, challenges remain. Errors in sensor accuracy, latency in force transmission, and a limited force feedback range introduce secondary errors that can distort operator perception and even mislead the operator during delicate navigation.

In addition to establishing feedback systems that mimic proximal forces, researchers have also explored intelligent feedback systems. By leveraging force and displacement feedback from the slave robot, deep learning algorithms can be employed to train the system for more precise catheter delivery control,^148^ while simultaneously reducing contact forces on the vessel wall.^149^ By mapping measured forces directly to operator feedback, such systems aim to bypass intermediate transmission stages, reducing error accumulation, and enabling reliable closed-loop control. Despite the promising capabilities of deep learning models, their generalization and robustness remain significant challenges, particularly due to their black-box nature and lack of interpretability. In contrast to conventional feedback strategies, recent human-robot interaction studies have explored the integration of augmented reality (AR) to operate robotic systems.^150^ This approach preserves the operator’s direct sensing to force feedback while overlaying real-time 3D visual feedback, thereby enhancing spatial perception and control accuracy. However, current AR technology exhibits projection errors of approximately 1 mm, which is considerable relative to the narrow and delicate vascular environment, potentially introducing safety concerns. Therefore, while intelligent feedback systems represent a promising frontier, they require further technological refinement and experimental validation.

#### Distal force sensing

Distal forces primarily arise from direct contact or collision between the tip of the interventional robot (e.g., a catheter or a guidewire) and the vessel wall. These forces can be indirectly reflected as measurable values at the proximal end. However, because of confounding factors such as frictional resistance and blood viscosity along the robot, accurately inferring distal forces from proximal measurements alone is challenging. To overcome this limitation and avoid excessive forces that may damage delicate vascular tissues, various distal force-sensing technologies have been developed and applied.^40^^,^^42^^,^^134^^,^^151^

Since traditional computational methods cannot directly infer distal forces from proximal measurements and robotic kinematic states, deep learning-based methods have been increasingly explored to estimate distal force values.^152^^,^^153^ These studies demonstrate promising potential for improving the force-sensing capabilities of EIRSs. Nevertheless, these approaches still require validation under more realistic experimental conditions and biological testing to ensure their robustness and generalizability. Given that the distal force-sensing directly involves interaction with the vascular environment, sensors play a pivotal role in ensuring patient safety, enabling real-time feedback, and providing a quantitative basis for control decisions. Thus, the development of distal force-sensing technologies must address three fundamental challenges.

(1) ensuring safe operation by detecting and limiting excessive contact forces,
(2) achieving high temporal responsiveness to dynamic surgical environments,
(3) establishing clear judgment criteria for robotic decision-making.

#### Intravascular ultrasound

In the field of interface sensing, in addition to force sensing, visual sensing technologies—such as intravascular ultrasound (IVUS) (see Figure 9A)—also play a crucial role. Real-time acquisition of 3D endovascular information is essential. This is because current *in vitro* world map sensing methods can provide only 2D vascular information intraoperatively and in real time, lacking depth. IVUS technology overcomes several limitations of conventional external US sensing by employing high-frequency 2D imaging with transducers located at the distal end of the interventional robot. This approach avoids interference from intestinal gas and preserves the inherent advantages of US technology. However, its application in practical robotic settings remains limited, and challenges such as low image resolution have yet to be fully resolved.^129^^,^^154^

#### Optical coherence tomography

Another technique for *in vivo* imaging is optical coherence tomography (OCT) (see Figure 9C), which employs rotational scanning using a laser positioned at the tip of the interventional robot. OCT offers several advantages, including a simple hardware design, high scanning frequency and resolution, and relatively low cost.^77^ These features enable the rapid and accurate acquisition of endovascular environmental information. However, because it relies on optical signal acquisition, neither frequency-domain nor time-domain OCT can effectively penetrate blood.^155^^,^^156^ As a result, its scanning range is limited in blood-filled environments, making it unsuitable for real-time use in EIRSs. Consequently, most surgical procedures using OCT require temporary cessation of blood flow to clear the field of view, which reduces surgical efficiency and increases procedural complexity and risk.

Overall, in the field of interface sensing, techniques capable of acquiring 3D information have been developed. However, the image quality and data acquisition capabilities of these sensing methods still require significant improvement. In addition, one promising approach to enhancing sensory input is the integration of haptic feedback to simulate a realistic surgical environment for the decision-making system. Nevertheless, ensuring the accuracy and fidelity of the acquired data, while simultaneously delivering effective and responsive feedback, remains a critical challenge that demands further research.

#### Control module

In conventional manual surgery, the physician plays a central role in both decision-making and control. In a master-slave EIRS, the physician issues motion commands on the master side based on integrated feedback from various sensing modalities. The robotic system on the slave side then executes these commands to navigate to the target site. This arrangement effectively shifts much of the control function to the robotic system, with the physician assuming a supervisory role focused on high-level decision-making and oversight. However, this separation of the control module introduces challenges in human-robot interaction within EIRSs, particularly regarding the seamless integration of physician expertise with automated control mechanisms.

#### Traditional method

To achieve precise control in complex vascular scenarios, researchers have proposed various control algorithms. Early master-slave interventional robotic systems employed open-loop control algorithms.^157^ An open-loop controller has no feedback path, and the system response is fast. However, due to poor disturbance rejection and limited environmental adaptability, its control accuracy is low. In order to improve control accuracy, closed-loop control algorithms were gradually adopted.^28^

**Figure 10:**
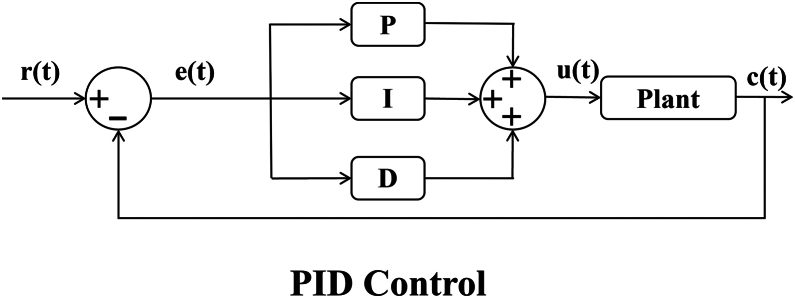
Schematic diagrams of Classic PID controller

The proportional-integral-derivative (PID) control algorithm is one of the most commonly used closed-loop control methods (see Figure 10). It adjusts the system inputs proportionally, integrally, and differentially according to the magnitude of the error.^158^ The PID algorithm is favored for its simplicity, ease of implementation, and tunable parameters, making it suitable for many control tasks. Nonetheless, its primary limitation lies in its reliance on linear models, which leads to suboptimal performance in systems with nonlinear dynamics, time-varying characteristics, or high uncertainty. Moreover, PID control lacks adaptability to system variations or external disturbances, and its parameter tuning often depends on empirical knowledge and iterative adjustment.^159^^,^^160^

To address the limitations of the traditional PID algorithm, researchers have explored adaptive control strategies, such as the fuzzy PID algorithm^161^ and the sliding mode control (SMC) algorithm.^162^ The fuzzy PID algorithm integrates fuzzy control theory with conventional PID control, allowing dynamic adjustment of the proportional, integral, and derivative parameters in response to changes in the system environment.^163^ This approach enhances adaptability and improves control performance under varying conditions. SMC is a nonlinear control strategy that achieves system stability by designing a sliding mode surface along which the system state slides. Both fuzzy PID and SMC offer advantages in handling system nonlinearities, uncertainties, and external disturbances. However, several challenges remain. These methods still depend heavily on expert knowledge and experience for controller design and parameter tuning. In the case of fuzzy PID control, the construction of the fuzzy rule base is particularly demanding, and it is difficult to guarantee that all possible control scenarios are adequately covered. As for SMC, its inherent switching behavior can lead to a phenomenon known as “chattering,” where the system state oscillates around the sliding surface rather than converging smoothly to the equilibrium point. This chattering effect can degrade control accuracy and compromise system stability.

#### Learning-based method

In recent years, with the development of learning-based technologies, various control strategies leveraging deep learning and reinforcement learning have emerged.^19^^,^^164^^,^^165^ These approaches have demonstrated superior control performance compared to traditional algorithms. Neural network models have a strong learning ability, can approximate complex nonlinear functions, and have a strong modeling ability for complex nonlinear time-varying systems. At the same time, they have the ability of adaptive adjustment, which can automatically adjust the control strategy according to the changes in the system environment.

**Figure 11:**
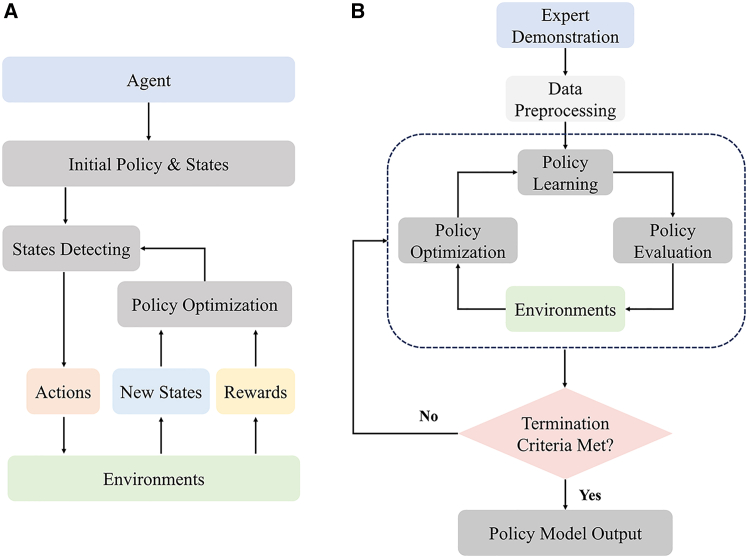
Learning-based methods for interventional robot navigation (A) Reinforcement learning improves navigation performance by learning optimal control strategies through trial-and-error interaction with the environment. (B) Imitation learning acquires navigation skills by learning from expert demonstrations, enabling efficient policy initialization and smooth trajectory generation.

Imitation learning (IL) (see Figure 11) offers an efficient approach for training interventional robots by enabling them to learn navigation strategies from expert demonstrations. Early studies employed Gaussian mixture models (GMM) and hierarchical hidden Markov models (H-HMM) to generate smooth motion trajectories. These trajectories were further optimized using techniques, such as dynamic time warping (DTW) and Gaussian mixture regression (GMR).^166^^,^^167^ One research team proposed an IL-based method that integrates non-rigid alignment with GMM to map catheter tip trajectories to various anatomical structures.^168^ They also introduced an approach combining path integral reinforcement learning (PI^2^) with dynamic motion primitives (DMP) to optimize catheter movements within a simulation environment.^169^ Subsequently, the same team proposed a generative adversarial imitation learning (GAIL)-based approach combining generative adversarial networks (GAN) and proximal policy optimization (PPO) to refine catheter navigation strategies across different vascular models.^65^ More recently, a neural network architecture combining an expert navigation network (ENN) and multimodal inputs was proposed and validated in the open-source simulator CathSim, further advancing the development of IL in practical applications.^170^ Additionally, by utilizing the Simulation open framework architecture (SOFA) and integrating inverse reinforcement learning (IRL) with the soft actor-critic (SAC) algorithm, researchers have demonstrated the ability to learn navigation strategies directly from expert demonstrations.^171^^,^^172^

In addition, significant progress has been made in applying deep learning techniques to interventional robot navigation. Some researchers have proposed using back propagation (BP) neural networks to tune the parameters of PID controllers, thereby enhancing control performance.^173^ A sample-efficient method based on deep reinforcement learning (DRL) combined with an episodic policy transfer strategy has also been introduced, which integrates with PID control for motion regulation of an EIRS.^163^ To reduce contact force between the catheter and the vessel wall, a compliant motion control algorithm based on long short-term memory (LSTM) networks was proposed.^165^ Another study employed a neural network model incorporating both LSTM and gated recurrent units (GRU) to compensate for positional errors in an EIRS,^164^ further demonstrating the potential of LSTM-based architectures for motion control in complex environments. Moreover, the combination of recurrent neural networks (RNN) with the SAC algorithm has been used to train guidewire controllers capable of navigating through various aortic arch geometries.^66^

In recent years, DRL (see Figure 11) has achieved significant progress in the field of EIRSs’ navigation. Deep Q-learning (DQN) and its variants have shown strong performance in navigation tasks involving discrete action spaces. One study applied a reinforcement learning approach based on Dueling DQN to achieve autonomous control of interventional robots.^64^ The model’s generalization capability was enhanced by introducing randomized noise to reduce the gap between simulated and real-world environments. Some researchers have proposed the rainbow algorithm (combining various improvements of DQN) to realize autonomous navigation.^19^ The training efficiency was further improved by incorporating segmented learning and expert demonstration data, while task-specific state and reward functions were designed using focus windows and subgoals. Additionally, one research has trained a catheter navigation model using DQN within the SOFA simulation environment, validating its applicability in complex anatomical structures.^174^ Within the SOFA framework, an RL-based strategy integrating PPO, generalized advantage estimation (GAE), and domain randomization (DR) was implemented to realize autonomous guidewire navigation with sim2real transfer capability.^175^

Deep deterministic policy gradient (DDPG) algorithms are widely adopted in interventional robot navigation tasks due to their excellent performance in a continuous action space. One research team trained both DQN and DDPG models, accelerating the guidewire navigation process using hindsight experience replay (HER) and human demonstration techniques to achieve effective autonomous control of interventional robots.^18^^,^^20^^,^^22^ More recent research implemented DDPG-based catheter navigation within the CathSim virtual environment, further demonstrating the potential of this algorithm for practical applications.^176^

The asynchronous advantageous actor-critic (A3C) algorithm is gradually being used in interventional robot navigation tasks, owing to its efficient parallel training capabilities. Some researchers have developed an A3C-based DRL framework and simulated catheter navigation in the Unity engine, demonstrating its potential for real-time applications.^177^ Another study implemented A3C-based autonomous catheter navigation in a SOFA simulation environment, validating its effectiveness in handling high-dimensional state spaces.^178^ More recently, a study team proposed a zero-shot reinforcement learning strategy combined with the SAC algorithm.^46^^,^^179^ This approach enables navigation through previously unseen vascular anatomical structures, opening new directions for the application of reinforcement learning in interventional robotics.

Recent studies have highlighted the potential of transformer architectures and large language models (LLMs) to improve perception-decision coupling in endovascular robotic navigation. Huang et al. introduced HPformer, a transformer-based high-frequency predictive model that fuses inertial and binocular-vision data.^180^ It enables 200 Hz visual-haptic feedback for virtual surgical navigation with low positional and directional error, thereby enhancing real-time motion prediction. Building on the transformer paradigm, Jianu et al. developed SplineFormer, which predicts B-spline control points and knots from fluoroscopic images to represent guidewire geometry.^181^ This method provides an interpretable control state and achieves fully autonomous navigation on a physical robotic platform. Extending beyond pure transformer inference, Yao et al. proposed a multi-agent fuzzy reinforcement learning framework guided by LLMs.^182^ It integrates Takagi-Sugeno fuzzy constraints into a centralized-training/decentralized-execution scheme, enabling cooperative guidewire-catheter navigation in SOFA-based 3D vascular simulations. This approach improves success rates and path efficiency compared with conventional reinforcement learning baselines. Collectively, these studies demonstrate how transformer-based representations and LLM-driven policy reasoning can strengthen decision-making for autonomous endovascular navigation, while underscoring the need for further validation in clinically realistic, real-time environments.

To provide a rigorous, cross-method performance assessment of the diverse learning-based strategies described above, representative studies were comprehensively compared across core performance metrics. This evaluation focuses on control accuracy, response delay, learning stability, and robustness/generalization, which are essential for assessing the real-world potential of learning-based EIRS control strategies. Table 2 presents an integrated quantitative summary of these studies, consolidating key metrics and experimental contexts across IL, deep learning, DRL, and transformer/LLM-based approaches, thereby enabling direct and transparent comparison of their respective strengths and limitations.

**Table 2:** Extended quantitative comparison of learning-based control and navigation methods for EIRSs

Reference	Method/Model	Platform	Environment Type	Performance Metrics			
				Success rate	Operation time	Contact force	Remarks
Chi et al.^65^	GAIL (GAN + PPO)	phantoms	dynamic, flexible	94.4% (Aortic arch-I), 72.2% (Aortic arch-II)	52.1 ± 9.9 s (Aortic arch-I), 172.3 ± 18 s (Aortic arch-II)	mean force: 0.34 ± 0.07N (Aortic arch-I), 0.18 ± 0.02N (Aortic arch-II)	path length: 55.7 ± 9.4 mm (Aortic arch-I), 201.6 ± 45.5 mm (Aortic arch-II) mean speed: 1.03 ± 0.03 mm/s (Aortic arch-I), 1.16 ± 0.26 mm/s (Aortic arch-II)
Jianu et al.^170^	IL (ENN)	virtual environments	dynamic, rigid	100% (BCA), 100% (LCCA)	episode length: 36.88 ± 2.4 steps (BCA), 33.77 ± 5.33 steps (LCCA)	2.33 ± 0.18N (BCA), 2.26 ± 0.33N (LCCA)	path length: 15.78 ± 0.17 cm (BCA), 14.85 ± 0.79 cm (LCCA); safety: 45 ± 5% (BCA), 45 ± 5% (LCCA); SPL: 99% (BCA), 100% (LCCA)
Robertshaw et al.^171^	IRL + SAC	virtual environments	static, rigid	95% (single-device tracking), 96% (dual-device tracking)	22.5 s (single-device tracking), 24.9 s (dual-device tracking)	–	path ratio: 98.7% (single-device tracking), 98.9% (dual-device tracking)
Robertshaw et al.^172^	SAC + IRL	virtual environments	static, flexible	96% (unseen)	7.0 s (training time 48 h)	mean force: 0.24 N (below 1.5 N rupture threshold)	path ratio: 94.6 ± 7.3%
Omisore et al.^163^	DRL + PID	virtual environments	dynamic, flexible	–	–	–	max navigation error: 0.004 mm; RMSE: 0.007 mm
Karstensen et al.^66^	RNN + SAC	virtual environments	dynamic, flexible	75.0% (after 29200 episodes)	–	–	average translation speed: 30.1 mm/s; Average trajectory length: 499.1 mm
You et al.^64^	Dueling DQN	virtual environments + phantoms	virtual: static, rigid; phantom: static, rigid	96%–98% (virtual); 73%–87% (phantom)	–	–	average distance: 4.29 ± 0.45–4.44 ± 0.72 mm (Virtual); 6.58 ± 5.60–4.70 ± 1.59 mm (phantom)
Kweon et al.^19^	Rainbow	phantoms	dynamic, rigid	98% (2D), 99% (3D)	–	–	–
Yao et al.^175^	PPO + GAE + DR	virtual environments + phantoms	virtual: static, rigid; phantom: static, flexible	90% (virtual); 100% (phantom)	5.14–7.63 s (virtual); 53.0 ± 5.1 s (phantom)	–	mean error: 2.30 ± 0.43 mm; RMSE: 2.50 ± 0.24 mm
Karstensen et al.^18^	DDPG + HER	phantoms	static, rigid	96% (52500 training episodes), improved to 100% (Using bifurcation coordinates as interim targets)	–	–	failures: categorized as high-level (path planning) vs. low-level (branch maneuvering); only high-level failures remained after full training
Behr et al.^20^	DQN & DDPG + HER/HD	virtual environments + phantoms	virtual: static, rigid; phantom: static, rigid	86.5% (DQN), 89.6% (DDPG)	training time: DDPG 9 h (+HER/HD, 11000 episodes) vs. plain DDPG 12.3 h (15000 episodes)	–	–
Karstensen et al.^22^	DDPG + HER	virtual environments + *Ex vivo* porcine liver	virtual: static, rigid; *Ex vivo*: dynamic, flexible	100% (virtual); 30% (*Ex vivo*)	*Ex vivo*: 14-18 s (robot) vs. 12-17 s (manual)	–	*Ex vivo* failure due to wrong branch 33.3%, entanglement 36.6%
Tian et al.^176^	DDPG	virtual environments	static, rigid	80% (Aortic arch-I), 100% (Aortic arch-II)	12 ± 1.2 s (Aortic arch-I), 4 ± 0.8 s (Aortic arch-II)	mean contact: force ∼0.002 N; max contact force: 0.079 ± 0.027 N (Aortic arch-I), 0.017 ± 0.002N (Aortic arch-II)	–
Meng et al.^178^	A3C	virtual environments	static, rigid	–	68.61 s (training) vs. 97.35 s (manual)	maximum contact force: 22.15 mN (training) vs. 43.27 mN (manual)	–
Scarponi et al.^46^	Zero-shot RL + SAC	virtual environments	static, rigid	95%	training time: 2 h/120000 steps	–	–
Scarponi et al.^179^	SAC	virtual environments	dynamic, flexible	97% (Heart) 93% (Liver)	training time: 6 h	–	–
Huang et al.^180^	HPformer (Transformer-based)	virtual environments	static, flexible	–	–	–	MAE: 0.0253 mm; RMSE: 0.0398 mm; navigation frequency: 200Hz
Jianu et al.^181^	SplineFormer (Transformer-based)	phantoms	dynamic, flexible	50%	150 ± 45.6 s	–	–
Yao et al.^182^	MAFRL (LLMs-guided)	virtual environments	static, rigid	70%–95%	average steps: 214 - 115 steps	–	path ration: 0.72–0.86

This table integrates critical performance indicators and experimental contexts reported in the literature, providing a unified benchmark for assessing algorithmic robustness, clinical applicability, and future research directions. “—” means no related data in the references. “∼” means estimated values from the references. “MAE” means mean absolute error. “RMSE” means root-mean-square error. “BCA” means brachiocephalic artery. “LCCA” means brachiocephalic artery.

From this comprehensive analysis across IL, DL, DRL, and transformer/LLMs-based methods for endovascular catheter and guidewire control, several trends emerge.

(1) Control performance and responsiveness: learning-based strategies—ranging from IL to advanced LLMs-based—consistently achieve high navigation success (often ≥90%) in virtual and phantom environments, with millimeter-level accuracy and sub-5 ms feedback, validating their technical maturity for precise endovascular manipulation.
(2) Learning stability and adaptability: curriculum learning, HER, and reward shaping effectively stabilize convergence and reduce training variance, while transformer-based predictors and hybrid controllers enhance sample efficiency and real-time tracking.
(3) Robustness and generalization: DR and zero-shot policies improve generalization to unseen anatomies, yet substantial performance decline in *ex vivo* and early clinical tests reveals an enduring sim2real gap and highlights the need for richer, standardized datasets across phantoms, *ex vivo* tissues, and clinical contexts.

Collectively, these findings underscore the clinical promise of learning-driven autonomous catheter and guidewire control, while identifying limited cross-domain data and incomplete sim2real transfer as key obstacles to safe and reproducible clinical translation. Building upon these insights, it is important to further examine the broader technical and practical challenges that currently hinder robust clinical deployment.

While an increasing number of researchers are applying DRL and LLMs to enhance autonomous control for EIRSs, several critical challenges persist. First, high-quality datasets are essential for learning-based approaches. However, constructing such datasets is hampered by the tedious and time-consuming calibration and the difficulty of acquiring reliable clinical data from surgical settings, constrained by safety, ethical considerations, and variability in data quality. Second, the limited generalization ability of current models in complex and dynamic surgical environments often results in unstable real-world performance. Third, the inherent lack of interpretability makes it difficult for physicians to trust the models’ decision-making, thereby increasing the potential risk during surgical procedures.

#### Data acquisition platform

**Figure 12:**
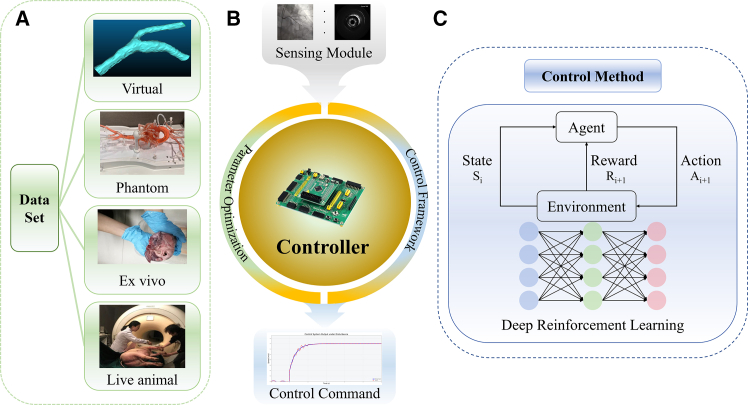
Diagram of an end-to-end intelligent control model (A) Dataset. Training and validation data are gathered from virtual environments, phantoms, *ex vivo* tissues, and live animals, enabling safe, scalable data collection when clinical data are limited. (B) Pipeline and operation logic. During operation, the sensing module provides image and auxiliary signals that are fused into task states; the learning model is trained to improve policy competence and then outputs optimal control commands. (C) Training methods (examples). Multiple learning paradigms can be used to train the end-to-end controller; DRL is illustrated here as a representative method, but IL, offline RL, and hybrid/fusion strategies are also applicable. Takeaway: The figure emphasizes how multi-source data, unified perception-decision-control, and learning-based methods jointly enable autonomous EIRS beyond conventional master-slave designs. Figure reproduced with permission from: a, *ex vivo* and live animal.^82^

Adaptive decision-making and control are a critical part of autonomous EIRSs. To achieve an autonomous robot system, it is necessary to develop a neural network model that can match or even surpass the capabilities of expert physicians. However, most existing platforms still rely on master-slave designs, where cooperative control between the operator and the robot inherently limits the system’s autonomy. To overcome this limitation, an alternative approach involves integrating decision-making and control into a unified, end-to-end learning model, analogous to the role of a surgeon in traditional manual procedures (as illustrated in Figure 12). Since the training of large models requires a large dataset, there are many challenges associated with building datasets collected in the clinic. Due to ethical and practical constraints, it is often impossible to collect sufficient high-quality data directly from clinical procedures. As a result, the use of simulators has become an indispensable strategy in the autonomy of EIRSs.

**Figure 13:**
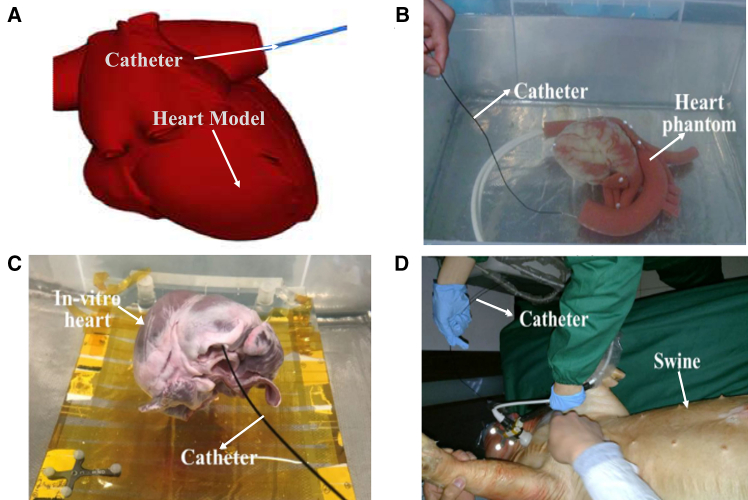
Data sources for training and validation of EIRS autonomy (A) Virtual environments provide scalable, controllable anatomy, and imaging. (B) Phantoms enable repeatable bench testing, allowing controllable flow, geometry, and instrumentation. (C) *Ex vivo* tissues capture realistic biomechanics for device-tissue interaction. (D) Live animals approximate clinical hemodynamics for protocol validation and safety assessment. Figure reproduced with permission from: A^64^; B, C, and D.^82^

Simulated environments provide a flexible and scalable approach for generating diverse, high-quality datasets, thereby supporting the development and validation of autonomous EIRSs. In the context of physician training, endovascular skills are typically acquired through four levels of simulators: phantoms, animals, virtual reality systems, and human cadavers.^183^ However, the simulators used for robotic system training differ from those designed for human learning. Taking into account both fidelity and cost, robotic training simulators can be classified into four categories: virtual environments, phantoms, *ex vivo* tissues, and live animals (as illustrated in Figure 13). Currently, most research relies on virtual environments and phantoms for algorithm training, while *ex vivo* tissues and live animals are primarily used for system validation and performance testing. In this regard, it is worth noting that live animal models employed in robotic studies are often integrated into training frameworks already approved for surgical fellow education. This practice ensures compliance with established ethical standards, optimizes resource utilization, and enhances translational relevance by paralleling conventional surgical training environments.

#### Virtual environments

In virtual environments, vascular interventional robots and vascular anatomies are simulated using mathematical and physical principles. Meng et al. developed a custom simulator based on the open-source SOFA framework and cantilever beam dynamics, proposing a Timoshenko beam theory-based modeling approach for guidewires that allows stiffness and mass properties to be adjusted through parameter tuning.^178^ Building on the same framework, Yao et al. introduced Sim4EndoR, a reinforcement learning-centered 3D simulation platform that incorporates anatomy-aware reward functions and demonstrates successful sim2real transfer for autonomous guidewire navigation.^184^ You et al. employed the Unity engine to model interactions between a catheter and a heart, focusing on collision behaviors; however, their approach only considered collision events while neglecting body deformation, thereby limiting its realism.^64^

Jianu et al. presented CathSim, an open-source simulator built on MuJoCo, enabling high-fidelity modeling of catheters and aortic anatomies with real-time force sensing, and offering a standardized platform for reinforcement learning-based algorithm development and benchmarking in autonomous navigation.^185^ Extending this platform, Tian et al. constructed a discretized catheter model composed of 100 linked segments to train and evaluate DDPG and PPO algorithms; however, since control was applied at the distal tip rather than the proximal end, the design fails to replicate actual clinical practice and thus limits its fidelity for practical applications.^176^

Complementing these domain-specific simulators, Schmidgall et al. introduced Surgical Gym, an open-source GPU-resident platform built on Isaac Gym that executes physics simulation and reinforcement learning entirely on the GPU.^186^ This framework provides markedly faster simulation and policy training than previous surgical-learning suites, enabling scalable and reproducible policy development. However, its current benchmarks remain primarily rigid-body and simulation-only, underscoring the need for soft-tissue modeling and hardware validation before clinical translation.

#### Phantoms

To evaluate the performance of interventional robots, researchers have also conducted experiments using vascular phantoms. These physical phantoms vary depending on the specific research objectives. Some studies have utilized 2D vascular models to test EIRSs,^18^^,^^19^^,^^20^ while others have employed 3D vascular models to achieve higher anatomical realism.^187^^,^^188^^,^^189^ Given the substantial anatomical variability in human vasculature, training or testing with a single vascular model often lacks generalizability to real-world surgical scenarios. However, fabricating multiple vascular molds to represent different anatomies is both time-consuming and costly. Virtual simulation environments offer a practical solution to this challenge, allowing for the rapid generation and modification of diverse vascular structures without the need for physical reconstruction.

#### *Ex vivo* tissues and live animals

To achieve better transferability of algorithms and systems to real-world surgical scenarios, researchers are continually working to replicate the clinical environment more accurately. Chen et al. validated their system through a series of experiments conducted in increasingly realistic settings, including a heart phantom, a water tank environment, *ex vivo* cardiovascular tissue, and live pig models.^82^ Gopesh et al. evaluated the microcatheter they developed using an *in vitro* silicone vascular model and further verified its embolic coil delivery performance in a live pig model.^190^ Similar to the phantom field, due to the significant anatomical variability among blood vessels, training on a single model often fails to ensure sufficient algorithm robustness.

Although real simulators offer higher fidelity, they are constrained by ethical and safety concerns, making them unsuitable for large-scale data generation or algorithm training. Therefore, virtual environments are widely favored in research due to their low cost, safety, and scalability—particularly given the substantial volume of training data required for deep learning algorithms. However, the domain gap between virtual and real environments remains a major challenge, and effectively addressing the sim2real transfer problem is critical.

#### The limitations in sim2real

Recent advancements in AI, particularly in deep learning and reinforcement learning, have demonstrated promising control performance when trained in virtual simulated environments. However, the ability of these virtual environments to generalize effectively to real surgical scenarios remains a significant challenge. Many real-world phenomena are difficult to replicate accurately in simulations. The major limitations include.

(1) Catheter/guidewire modeling error: various modeling techniques have been proposed for simulating catheter and guidewire behavior, including finite element models (FEM),^191^ beam-like FEM models,^192^ and mass-spring models.^193^ However, modeling errors persist due to multiple sources of uncertainty, such as manual setup variability, manufacturing tolerances, material property inconsistencies, and grid discretization errors. Furthermore, accurately capturing the coupling interaction between the catheter and the guidewire in a simulation remains extremely difficult, leading to discrepancies between simulated and actual performance.
(2) Environmental error: significant errors arise from discrepancies between the simulated and real surgical environments. These include vascular deformation caused by friction and collisions between the interventional device and the vascular wall, as well as dynamic physiological changes such as blood flow, pulsation, cardiac motion, and vessel occlusion. Such factors are difficult to model with high fidelity, resulting in environmental mismatch.
(3) Real-time: simulated systems typically operate in a step-by-step manner, which differs from the real-time, dynamic, changeable natures of actual surgical procedures. Achieving real-time simulation of catheter and guidewire behavior is particularly challenging. The simulators must perform complex tasks such as collision detection and response, as well as compute optimal motion paths based on the device’s dynamic characteristics. These computational demands pose a significant burden on real-time performance. Improving the simulator’s responsiveness while ensuring accuracy and stability remains a critical area for further research.

Regardless of the type of simulator used, a significant gap still exists between simulated environments and real surgical scenarios. The sim2real transfer problem remains a major challenge, especially in the context of endovascular intervention. Most current research addressing this issue is concentrated in the field of DRL for general robotics. Common techniques have been explored, including DR, domain adaptation, IL, meta-learning, and knowledge distillation. However, studies focusing on sim2real transfer for EIRSs are still limited. In one such study,^194^ the researchers applied data augmentation techniques to introduce randomization into the simulated data, enabling the trained neural network to generalize better to real-world conditions. A limitation of this approach, however, is that it treats a physical vascular phantom as the real-world reference, which may still differ significantly from actual patient anatomy and physiology.

### Execution module

#### Interventional robot mechanism according to the paradigm used by surgeons

**Figure 14:**
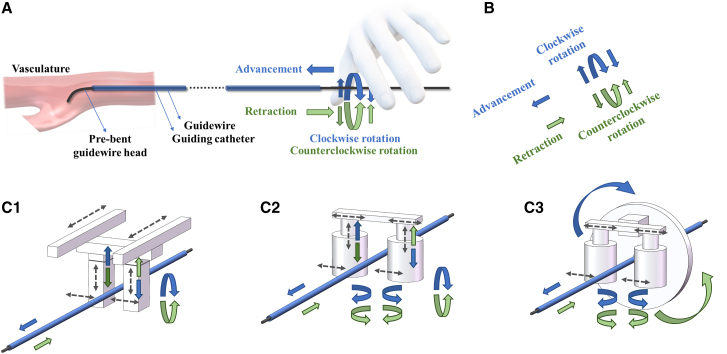
Mechanisms for guidewire translation and rotation (A) Manual guidewire manipulation by the surgeon, where coordinated pinching and rubbing motions of the fingers generate axial advancement and directional steering. (B) Abstraction of the manual manipulation paradigm into three core actions—clamping, translation, and rotation—that define the essential requirements for mechanical implementation. (C1) Bionic clamp fingers. Two opposing fingers grip the guidewire. Axial reciprocation along the *x* axis advances or retracts the guidewire; differential linear motion along the *z* axis applies tangential friction to induce tip rotation. (C2) Dual friction wheels (with differential lateral motion). A pair of counter-rotating wheels clamp the guidewire to generate axial translation; controlled opposite linear motion along the *z* axis creates differential friction that produces rotation. (C3) Dual friction wheels with rotary base. The counter-rotating wheels translate the guidewire as in (C2), while a rotating disk drives the entire assembly to rotate about the guidewire axis, enabling continuous or high-authority rotational manipulation.

Manipulating guidewires and catheters through blood vessels requires surgeons to skillfully coordinate their thumbs and index fingers.^195^ Typically, the thumb and index finger pinch the guidewire or catheter, and a gentle push on the proximal portion advances the distal tip along the vessel path. When encountering a vascular bifurcation, the surgeon performs a coordinated rubbing motion—moving the fingers up and down relative to each other—to steer the pre-bent tip of the guidewire into the desired branch. Figure 14A illustrates this manipulation and provides a force analysis of the interaction between the surgeon’s fingers and the device. Based on the operational patterns shown in Figure 14B, it can be deduced that at least three fundamental actions—clamping, translation, and rotation—are necessary for replicating the surgeon’s manual operation. These actions correspond to the 2° of freedom (2-DOF) required for intravascular tool navigation: axial movement and rotational control. Figure 14C classifies various actuation mechanisms used in EIRSs, particularly focusing on translational and rotational drive systems.

Among them, the gripper mechanism (c1) most closely mimics the human hand by achieving reciprocating motion through clamping and unclamping. It offers high reliability and precise control during guidewire advancement but is bulky and poses challenges for sterilization.^196^ The friction wheel mechanism (c2) translates the guidewire by clamping two wheels and rotating them counter-rotationally along the axis. It has the advantage of being compact and easier to sterilize, but may suffer from slippage, leading to reduced control accuracy.^197^ Rotational mechanisms include the bionic finger-claw mechanism (c1, c2) and the rotating disc mechanism (c3). The former replicates thumb-index finger rotation but is limited in rotation angle and precision.^158^ The rotating disc mechanism utilizes gears or timing belts to achieve high-precision rotation, but may interfere with translational delivery.^198^

Commercial EIRS platforms, such as the CorPath GRX and Magellan systems, combine these two basic mechanisms to enable full control of intravascular tools.^199^ In addition, novel actuation strategies are being explored to enhance mechanical performance. Shen et al. proposed a hybrid translational mechanism to improve guidewire delivery accuracy,^198^ while Choi et al. introduced a modified friction wheelset to mitigate slippage.^199^ Bian et al. developed a bionic finger claw system using friction wheels to emulate the surgeon’s hand movements.^196^ Wang et al. employed a multi-manipulator setup, where four manipulators independently controlled tool translation and rotation via wire ropes and motorized gears.^158^ Song et al. developed a novel endovascular robotic system equipped with dual mechanical arms, each carrying independent manipulators and double V-shaped grippers, enabling precise clamping, translation, and rotation of over-the-wire as well as rapid-exchange devices.^200^ Guo et al. developed a specialized endovascular robotic system combining dual mechanical arms and double V-shaped grippers with a third V-shaped gripper serving as a safety arm, enabling full remote device manipulation and markedly reducing operator radiation exposure.^201^ These studies not only advance the development of endovascular robots but also provide new possibilities for future clinical applications.

The design of interventional robots draws heavily on conventional catheter and guidewire manipulation techniques. Most clinically used catheters and guidewires rely on passive flexibility—meaning they cannot actively steer but instead depend on pre-shaped geometries. Surgeons typically advance a pre-shaped guidewire and manually rotate it at vascular bifurcations to steer it into the target vessel. This maneuver demands substantial expertise and fine motor control. A critical design requirement is minimizing backlash and friction in the proximal-to-distal actuation pathway, as these factors degrade the fidelity of motion transmission. Achieving precise motion transmission over long distances—from the proximal controller to the distal tip—remains a major challenge, especially given the stringent size constraints. This holds true whether actuation is mechanical (via the guidewire or catheter shaft) or external (e.g., magnetic fields).

**Figure 15:**
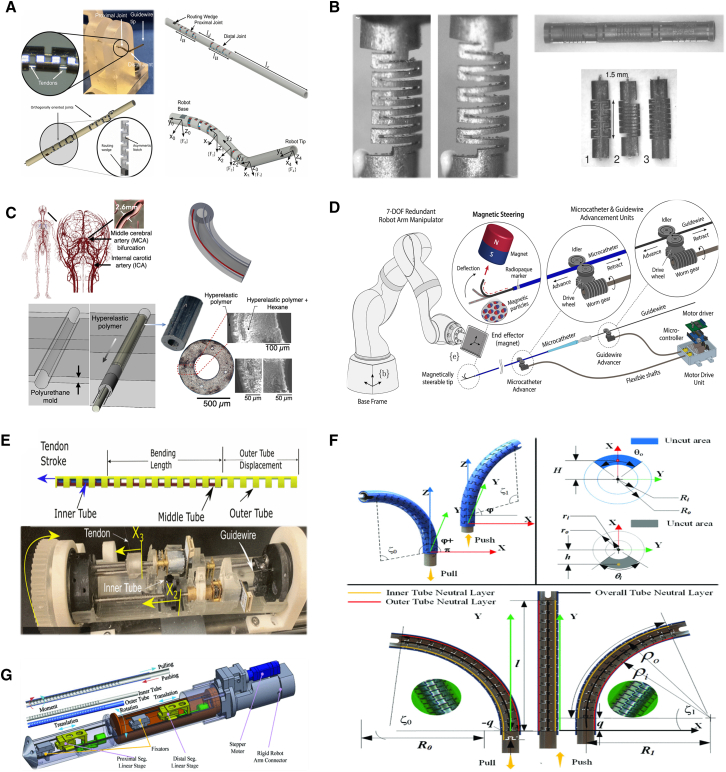
Various interventional robot mechanisms (A) Cable-actuated interventional robot.^203^ (B) Shape memory alloy-actuated interventional robot.^204^ (C) Pneumatic-hydraulic pressure-actuated interventional robot.^190^ (D) Magnetically actuated interventional robot.^205^ (E) Robotic guidewire system^71^: modified coil spring guidewire and compact actuation prototype. (F) Co-axial slender tubular robot^206^: bidirectional bending actuation modeling. (G) Cad model^206^: asymmetric co-axial tubes with push/pull and rotational actuation.

To address these challenges, the development of interventional robots with actively steerable tips has become essential. Such robots can enhance the success rate of vascular access and reduce the contact force between the vessel wall and the robot’s tip.^202^ Currently, there are four main types of steerable catheters (as shown in Figure 15): cable-actuated,^203^ shape memory alloy-actuated,^204^ pneumatic or hydraulic pressure-actuated,^190^ and magnetically actuated.^205^

#### Cable-actuated interventional robots

Cable-actuated interventional robots are relatively simple, cost-effective, and offer precise control. A key advantage of this design is its ability to bend the catheter tip more than 180°, which is particularly beneficial for navigating tortuous blood vessels. In cable-actuated systems, catheter bending is achieved by pulling on embedded cables, eliminating the need for a separate guidewire to provide directional control. Jung et al. developed a cable-driven parallel robot for X-ray-guided needle insertion.^207^ The system features a radiolucent end-effector and body-mounted base, enabling C-arm compatibility and motion synchronization with the patient. An 8-cable mechanism achieves 6-DoF motion with a virtual remote center of motion. Choset et al. developed a cable-actuated robotic system named CardioArm.^208^ This device consists of 50 rigid cylindrical segments connected in series via four drive cables and achieves omnidirectional bending through two motors that actuate the cables. Chitalia et al. proposed a laser-micromachined, cable-driven guidewire capable of precise orientation control.^203^ Deaton et al. designed a cable-driven catheter equipped with three adhered single-core FBG sensors, enabling real-time prediction and reconstruction of deformation and contact force at the tip.^202^ Yan et al. introduced a cable-actuated conduit based on a spring-driven, single-bar actuation structure that allows for precise attitude control.^209^

In cable-actuated interventional robots, the drive motors are typically placed at the proximal end—outside the patient’s body—to minimize the influence of motor vibration on distal tip control. Compared with other actuation strategies, cable-driven systems provide higher load capacity. Nevertheless, these designs face notable limitations. The need to prevent radial buckling of the push-pull cables necessitates a relatively rigid catheter shaft with substantial structural housing to ensure radial stiffness, thereby restricting their applicability in delicate anatomies such as cerebral vessels, where the vasculature is extremely small and fragile. Furthermore, size constraints and reduced distal flexibility may compromise maneuverability in tortuous pathways, while increasing numbers of drive units exacerbate cable-wall friction, introducing mechanical resistance that complicates motion control and reduces overall system responsiveness.

#### Shape memory alloy interventional robots

Shape memory alloy (SMA)-based interventional robots leverage the thermally induced deformation properties of SMA materials to achieve controlled bending movements. These robots are typically constructed from nickel-titanium (NiTi) alloys, which can return to their predefined shapes when exposed to specific temperatures. This property enables precise manipulation of the catheter by simply heating or cooling the SMA, allowing it to bend or straighten as needed within the vessel.

Several researchers have proposed innovative SMA-driven designs. Ikuta et al. developed an SMA interventional robot using a spring-based structure, with a multi-segmented head that simplifies control.^210^ To enhance safety and response speed, a cooling water pipe was incorporated along the central axis and stabilized with a spring. Haga et al. designed a widely adopted flexible conduit structure, incorporating three eccentrically positioned SMA actuators evenly spaced and secured to the links on either side.^211^ And they also developed a versatile SMA conduit with a 1.4 mm diameter that can bend, twist, and extend.^212^ Mineta et al. employed electrochemical pulse etching to fabricate S-shaped SMA actuators, which reduced the robot’s outer diameter to 0.95 mm while increasing the number of internal working channels.^213^ Chang et al. utilized a Z-shaped SMA actuator with a 1.5 mm outer diameter.^214^ Tung et al. introduced a novel actuator by laser-machining SMA tubing, enabling greater force and displacement than conventional SMA wire or spring actuators.^204^ A major challenge in SMA-driven systems is the large number of wires required to independently control each actuator. To address this, Lim et al. embedded integrated complementary metal oxide semiconductor circuitry into the linkage, allowing actuator switching and reducing the number of wires to just three.^215^

SMA-driven interventional robots represent a pioneering advancement in the field, offering active guiding capabilities that overcome the lack of rigidity in traditional guidewires. These robots are valued for their structural simplicity and rapid responsiveness, making them particularly suitable for applications that demand fast actuation and frequent shape changes. However, SMA-based systems also have notable limitations. They produce relatively low driving force and require precise temperature control to ensure consistent performance. Additionally, SMA materials are susceptible to fatigue, which can result in performance degradation over time. As a result, durability and maintainability should be carefully considered in the design process. Furthermore, their electrothermal actuation mechanism may cause patient discomfort or even tissue damage due to localized heating, with the most critical concern being coagulation of blood adjacent to heat sources. This risk is particularly pronounced in the neurovasculature and peripheral vasculature, where vessels are small and blood flow is limited, whereas in larger vessels, the higher mass flow rate mitigates the problem.^216^ Despite substantial progress in this area, challenges related to actuation temperature and structural flexibility remains and should be addressed to improve their practicality in procedures.

#### Pneumatically or hydraulically actuated interventional robots

Pneumatically or hydraulically actuated interventional robots utilize pressure changes—generated by air or fluid—to control the robot’s orientation. This type of actuation offers advantages such as simple structure, higher driving force, and strong stability. These robots often incorporate bellows-based structures, where variations in internal pressure cause the bellows to change their length accordingly, thereby altering the bending angle of the robot’s distal end. Ruzzu et al. proposed a pneumatically actuated catheter system that adjusts its tip diameter with airflow pressure, allowing precise positioning.^217^ Horovitz and Kosa developed an active conduit that combines hydraulic and thermal actuation principles, featuring a bellows-type elastic structure and circumferentially arranged resistance wires for directional bending control.^218^ Pourghodrat et al. introduced disposable fluidic actuators tailored for miniature *in vivo* surgical robots, emphasizing low-cost fabrication and single-use application to reduce infection risk and improve clinical safety.^219^ Gopesh et al. designed a hydraulically driven microcatheter with a superelastic body and hydrophilic coating, enabling smooth navigation in *ex vivo* and *in vivo* settings.^190^ Barnes et al. presented a pneumatically actuated soft robotic catheter tip with dual channels, controlled by an intuitive handheld device, which achieved rapid response and sub-millimeter positioning accuracy in phantom studies.^220^ Liang et al. developed a fully pneumatic, MRI-compatible robotic system for prostate interventions, employing 3° of freedom pneumatic stepper motors to achieve accurate needle guidance under MRI.^221^

A major concern with pneumatic actuation is the inherent risk of air embolism in the event of device failure, a safety issue significant enough to hinder regulatory approval for clinical use.^222^ Nevertheless, fluid-driven interventional robots as a whole provide distinct advantages, including high power density, strong distal controllability, and compliance that facilitates safe navigation through tortuous vessels. Despite these merits, critical challenges remain, particularly in microfabrication, sealing reliability, and the integration of complex tubing and valve systems within confined clinical environments.^190^ Moreover, thermo-hydraulic variants introduce additional risks related to thermal latency and safety.^218^ Overall, while these systems hold considerable promise, their successful clinical translation will depend on advances in microfabrication techniques, control integration, and adaptation to existing clinical workflows.

#### Magnetically controlled interventional robots

In recent years, with advances in technology, magnetic actuation—often referred to as magnetron technology—has emerged as a promising approach in the field of interventional catheter robotics. As demonstrated by Nelson et al. in their study, remotely magnetically navigated interventional robots use externally generated magnetic fields to guide and control embedded magnetic elements within the catheter, enabling highly precise navigation.^223^ This technique is particularly well-suited for complex vascular environments, where precise and flexible control is essential.

One of the most prominent systems in this domain is the Stereotaxis Niobe platform, which integrates robotic magnetic navigation for interventional procedures.^224^ It consists of dual external magnets, a fluoroscopy scanner, a visualization monitor, and an examination table. The system steers the catheter tip with high precision by generating a controlled magnetic field. Lalande et al. demonstrated *in vivo* navigation of magnetic guidewires using MRI-generated field gradients to steer ferromagnetic beads attached to the guidewire tip through the vasculature.^225^ Kim et al. developed a soft ferromagnetic robot based on a flexible polymer matrix embedded with uniformly dispersed ferromagnetic microparticles, enabling adaptable and responsive magnetic actuation for cerebrovascular intervention.^226^ Sa et al. proposed a separable and recombinable magnetic robot that integrates a delivery catheter with an untethered magnetic robot to achieve precise intravascular operations under external magnetic field control.^227^ Wei et al. designed a magnetically actuated continuum robot with NdFeB-PDMS material and a stiff core, and validated its endovascular performance through phantom navigation and magnetic deflection tests.^228^ Xu et al. developed a fully automatic magnetic guidewire steering system and introduced a double-loop SMC strategy that decouples velocity and direction control to achieve stable and accurate navigation in vascular models.^229^ In more recent work, Dreyfus et al. developed a helical magnetic robot with a rotational propulsion mechanism and an articulating magnetic tip for precise endovascular navigation.^230^ The design improves maneuverability, prevents structural buckling, and enables responsive control through external magnetic actuation, thereby significantly enhancing the precision and safety of endovascular interventions.

Magnetic actuation offers several advantages over conventional mechanical drive systems, particularly the ability to achieve non-contact control, which enhances the robot’s flexibility and maneuverability. Additionally, it enables structural simplification and potential miniaturization of robotic systems, making them more adaptable for complex vascular environments. However, several challenges hinder the practical implementation of magnetically actuated systems. These include difficulties in precisely controlling the magnetic field strength and distribution, selecting suitable magnetic materials, and ensuring their biocompatibility. Moreover, high system costs, technical complexity in integration, and limited compatibility with existing medical imaging modalities remain significant barriers to clinical translation.

In addition to the aforementioned types, a class of interventional robots on the concentric tube principle has gained increasing attention among researchers. Ravigopal et al. developed the COaxially Aligned STeerable (COAST) guidewire robot, consisting of three-layer superelastic nitinol tubes.^71^ The outer and middle layers are designed with unidirectional asymmetric cuts, and the inner layer is coated with parylene for water resistance. The device employs a tendon-driven motor system to enable precise bending and rotational control, supporting navigation through complex vascular or airway structures. Wang et al. developed the co-axial slender tubular (CAST) robot, featuring two asymmetric tubes offset by 180° and assembled coaxially, enabling bi-directional bending through the axial motion of the inner tube.^206^ The design incorporates a tendon groove mechanism to enhance structural rigidity, achieving a maximum positional error of 3 mm under open-loop control.

The development of minimally invasive catheterization robots has been propelled by a range of actuation strategies, each offering distinct advantages and facing specific limitations. Cable-driven interventional robots are notable for their high load capacity and operational stability. However, they face challenges related to bulkiness and increased difficulty in maneuvering within complex anatomical environments. SMA-driven robots exhibit promise due to their compact structure and rapid response, but their limited actuation force and reliance on precise temperature regulation constrain their practical utility. Pneumatically or hydraulically actuated systems can deliver substantial driving force and reliable performance. Whereas their widespread application is hindered by stringent requirements for high-precision manufacturing and controlled environments. Magnetically controlled robots offer the unique benefit of non-contact navigation, enhancing flexibility and miniaturization. Nevertheless, they are limited by the nonlinear characteristics of magnetic fields and the potential biocompatibility issues associated with ferromagnetic materials.

## Future expectations

Becoming fully autonomous will have a transformative impact on endovascular interventions, significantly improving the safety and precision of the procedure while substantially reducing the workload of the surgeon. In order to achieve this objective, it is necessary for the system to reach new heights in multimodal sensing, intelligent decision-making, and adaptive control. Specifically, the system must overcome the limitations of existing sensing technologies to provide more comprehensive and accurate 3D information feedback; develop models capable of simulating complex vascular environments to improve the system’s learning and adaptability; and develop highly autonomous interventional robots that can flexibly handle diverse surgical scenarios to achieve precise operations.

### Multimodal sensing and real-time fusion

In the part of robotic sensing, external imaging (e.g., X-ray fluoroscopy, MRI, and US) remains the clinical mainstay to construct the global view of anatomy and device pose. Well-documented constraints persist: radiation and contrast burden, 2D projection limitations in tortuous anatomies, device/EM constraints, or real-time latency in certain modalities.^231^ Global context alone is therefore insufficient for safe manipulation at the lumen wall. These constraints have driven interest in intraluminal sensing, notably IVUS-like sensors, to provide localized, blood-side information that complements the global, room-side view.

At the interface level, most current systems still estimate distal interaction from proximal or “external” measurements inside the drive mechanism. These signals, however, are susceptible to distortions from friction, backlash, and shaft compliance, which obscure the actual catheter-wall forces. Consequently, force decoupling becomes challenging, haptic feedback fidelity is compromised, and human-robot cooperation suffers. Contemporary reviews explicitly distinguish internal/distal versus external/proximal sensing and caution that proximal signals alone may not reflect in-vessel reality—arguing for new sensors and hybrid strategies.^7^^,^^10^ This motivates a shift toward distal sensing at the tip to directly capture contact forces and reduce decoupling error.

Recent prototypes demonstrate exactly this trajectory. Multi-core FBG sensors integrated at the catheter tip achieve 3D force measurement with millinewton-level resolution and clear decoupling between longitudinal and lateral loads, illustrating feasibility for clinical translation within compact designs.^232^ In parallel, temperature-compensated FBG distal sensors tailored for cardiac ablation have reported 0.01N resolution with simple, robust constructions amenable to scale-up, underscoring that miniature, distal, and harmless force sensing is becoming practical.^233^ Together, these results indicate that placing sensors at the operative tip can mitigate the distortion inherent to proximal measurements.

Beyond single-modality instruments, the field is moving toward multimodal sensing that fuses global-scale imaging (X-ray/MRI/US) with local-scale interface streams (IVUS/OCT/distal sensors) to build a higher-fidelity world model for navigation and collision avoidance. Recent reviews emphasize AI-enabled fusion and simulated/fused reality as key enablers precisely because they can integrate heterogeneous intraoperative data sources rather than relying on any single modality.^234^ A systematic review reinforces that decision support, automation, and multimodal imaging are converging areas, albeit many methods remain early-stage and need clinical validation—framing multimodal sensing as both necessary and timely.^235^

For fully or highly autonomous EIRSs, sensing must cover both global vessel-tree context and local device-tissue interaction. Evidence from autonomous navigation studies shows pipelines that already pair tracking or vision sensors with learned policies, supporting the premise that multiple data streams are operationally useful.^61^^,^^236^ Meanwhile, surveys of commercial robotic platforms document growing incorporation of machine vision, AI-assisted 3D reconstruction, and real-time guidance—signposts that clinical systems are incrementally embracing multimodal perception.^237^ In sum, future sensing for EIRS should prioritize low-risk, miniaturized distal sensors combined with internal imaging and AI fusion, thereby elevating data dimensionality and reliability while reducing dependence on brittle proximal feedback channels.

### Autonomous decision-making and adaptive control

To date, the autonomous decision-making capabilities of EIRSs in complex clinical scenarios remain limited. These systems still rely heavily on human operation, and most autonomous functions have been demonstrated primarily in virtual or phantom settings, leaving a considerable gap before full autonomy can be realized in clinical practice.^61^ Within this context, autonomous decision-making and adaptive control should be viewed as a coupled pipeline: the decision layer specifies task goals and safety/efficiency constraints, while the control layer translates them into precise motion and force regulation under intraoperative uncertainty. Contemporary interventional robots are still predominantly master-slave with bilateral control and safety strategies, underscoring the need to propagate high-level goals into verifiable motion/force bounds.^7^

A practical first step is to develop virtual environments that closely replicate human vasculature. Because the vascular system is highly dynamic and complex—shaped by blood flow and cardiac motion—simulation platforms should reflect adequate anatomical complexity and physiological realism. To ease the design burden and support different development stages, simulators can be organized into two complementary types: (1) foundational simulators for generalizable capabilities, and (2) task-specific simulators for advanced control and complex tasks in clinically relevant scenarios. This split mirrors practice in surgical autonomy research, where learning-based policies (e.g., reinforcement and IL) are matured in simulation before real-world exposure.^33^

Because virtual and real environments inevitably diverge, models should address the sim2real gap prior to clinical deployment. Simulations can incorporate substantial variability via DR—across perceptual data and dynamics—to improve generalization. While transfer-learning mechanisms, such as policy distillation and meta-learning, further support adaptation to real intraoperative conditions and reduce brittle behavior under distribution shifts. These strategies are consistent with broader roadmaps for embodied intelligence in endovascular robotics, which emphasize policy learning in simulation/digital-twin environments coupled with real-world feedback.^33^^,^^238^

While end-to-end learning offers advantages—including faster error correction and stronger performance in unfamiliar scenarios—it suffers from limited interpretability due to the black-box nature of neural networks. A modular, end-to-end framework can improve transparency by exposing auditable intermediate artifacts (e.g., planned paths, predicted risks, and uncertainty estimates), preserving the data-to-decision flow while making the rationale behind actions easier to inspect. This aligns with embodied-intelligence perspectives that prioritize explainable AI and human-in-the-loop oversight in endovascular settings.^238^ In parallel, generalist biomedical vision-language models demonstrate robust cross-task capability and may provide practical interfaces to surface planning rationales and task constraints for clinical review, though further validation is required.^239^

Within this architecture, future interventional robots are expected to achieve a higher degree of autonomy—independently navigating complex vascular networks, adapting their shape with flexibility, and precisely tracking target lesions—with the objective of minimizing vessel-wall contact and reducing tissue injury. Integrated AI algorithms can provide real-time analysis and decision support by predicting potential complications and optimizing surgical pathways, while adaptive control mechanisms adjust actions dynamically based on intraoperative feedback as conditions evolve. Nonetheless, current literature characterizes AI adoption as early-stage, with heterogeneous and non-standardized data—reinforcing the need for prospective validation before routine autonomy.^234^^,^^235^ Finally, standardized interfaces and workflow-aligned integration can streamline operations; overview-of-reviews highlights that integration is complex and will depend on guidelines, certification, clinician education, and governance frameworks to ensure safety and reliability.^235^ As experience accumulates, systems can iteratively refine their performance through prospective evaluation, enabling better decision-making and more effective adaptive control—ultimately achieving greater precision and higher success rates.

### Construction of the dataset and the evaluation benchmark

High-quality datasets are foundational for the performance of EIRSs, yet the community continues to face a pronounced shortage of publicly accessible data. As a result, many studies construct bespoke environments and collect data independently, increasing costs and complexity and making results difficult to compare across studies.^240^^,^^241^ Variability in acquisition, labeling, and data quality further confounds performance assessment; multi-institutional heterogeneity and privacy constraints also limit centralized curation, motivating federated or privacy-preserving learning strategies and tighter annotation standards.^242^^,^^243^ Establishing rich, diverse, and rigorously curated public datasets would therefore provide a common experimental foundation, strengthen reproducibility, and improve the comparability and reliability of reported findings. CathAction (a large-scale benchmark for endovascular procedure understanding with >500 k annotated frames for action/collision; ∼25 k segmentation masks) and Guide3D (a bi-planar X-ray dataset for 3D guidewire shape reconstruction with curated annotations and baselines) illustrate this direction.^240^^,^^241^ Such resources should document sensing modalities, patient/phantom characteristics, task definitions, annotation procedures, and transparent train/validation/test splits; they should also report inter-annotator agreement/adjudication and de-identification/consent policies.^240^^,^^241^^,^^242^ Taken together, standardized public datasets would not only reduce development friction but also lay the groundwork for credible, like-for-like benchmarking across tasks and platforms.^240^^,^^241^

In parallel with the data gap, there is no universally accepted benchmark for evaluating the autonomous capabilities of EIRSs. Most existing studies evaluate learning-based methods within custom experimental setups, demonstrating promise but limiting cross-study interpretability. A systematic review of AI-enabled endovascular autonomy similarly concluded that evidence remains at proof-of-concept with heterogeneous, non-standardized evaluations, underscoring the need for reference standards.^61^ A comprehensive, fair, and standardized evaluation framework is needed to quantify real-world performance and enable like-for-like comparisons not only among autonomous systems but also against traditionally manually operated or master-slave (teleoperated) robotic platforms. Where possible, evaluation should be organized around well-defined capability levels and image-guidance competencies.^60^ At minimum, benchmarks should specify representative tasks and operating conditions; define clear endpoints (e.g., success rate, time to target, wall-contact events, and path efficiency); and prescribe consistent reporting protocols (including confidence intervals, significance testing, external validation, or sequestered (hidden) test sets, and predefined analysis plans).^60^^,^^61^ Where appropriate, task definitions and metrics can be aligned with existing releases (e.g., CathAction, Guide3D) to facilitate like-for-like comparisons across methods.^240^^,^^241^ A unified scheme of this kind would support meaningful direct evaluation and clarify the true efficacy and limitations of autonomous systems relative to human experts while including safety-relevant endpoints (e.g., contrast volume, radiation dose, and complication rates). Reducing measurement error between studies would help accelerate method iteration toward safer and more effective clinical applications. EI-oriented reviews likewise highlight governance considerations (e.g., federated data sharing and explainable, human-in-the-loop evaluation pathways) that align with such benchmarking practice.^238^

### Design frontiers of EIRS: Smaller, softer, smarter

Compared with cardiovascular applications, robot-assisted neurointerventions face stricter physical and safety constraints: most intracranial target vessels are ≤1.5 mm in diameter and highly tortuous, so even minimal trauma can cause dissection or rupture; prolonged fluoroscopy further compounds patient and operator risk.^244^ Current tele-operated endovascular robots adapted from cardiology require specific software/hardware changes (e.g., anti-herniation cassettes, rotate-on-retract, and active device fixation), yet true distal haptics remain unavailable, which exacerbates kickback and wall-contact uncertainty in fragile cerebral arteries.^244^^,^^245^ Clinically, early neurovascular trials show feasibility but highlight the need for sub-millimeter precision and stable coaxial support over long access paths.^245^ Recent clinical data confirm robotic safety for aneurysm embolization and angiography, but also reveal overheads such as setup time and the enduring need for finer manipulation and sensing in the head and neck.^246^ Beyond hardware, “world map” limitations in sensing and control—imperfect environment models, sparse feedback, and latency—are amplified in the brain’s branching microvasculature, underscoring unmet needs in miniaturization, steerability, and force/shape sensing (e.g., FBG/EM) for reliable distal navigation. Contemporary reviews and trials converge on these pain points for neuroendovascular robotics, noting precision, radiation, and safety as dominant drivers for innovation.^247^^,^^248^^,^^249^

Taken together, these constraints define a concrete design brief for next-generation systems—richer perception beyond projective imaging, explicitly safety-aware decision policies, and neuro-tailored distal manipulation—which in turn motivates the opportunity directions below. In response, several complementary thrusts are emerging to meet neurovascular demands.

(1) Intelligent navigation and autonomy: reinforcement-learning controllers have recently achieved high success, force-aware two-device navigation from internal carotid artery to middle cerebral artery on unseen cerebral anatomies, explicitly minimizing tip forces—key for safety in fragile vessels.^172^
(2) Neuro-specific tele-operation platforms: a randomized multicenter trial of a dedicated cerebral robot (PANVIS-A) reported non-inferior success vs. manual angiography with a marked reduction in operator radiation, supporting clinical viability while motivating faster setup and finer distal control^246^; complementary studies of CorPath GRX for aneurysm treatment likewise demonstrated effectiveness and safety.^247^
(3) Soft, steerable, and variable-stiffness devices: soft-robotic microcatheters and steerable/variable-stiffness guidewires promise sub-millimeter tip placement in small, tortuous vessels, reducing wall stress and improving selection of distal branches.^190^ Magnetically actuated tips and next-gen magnetic navigation systems further enhance distal dexterity while improving energy efficiency and workspace control in the neurovascular domain.^250^^,^^251^^,^^252^

Collectively, these advances, together with microrobotics and continuum-robotics concepts, outline a path to a miniaturized, flexible, and AI-assisted system. These systems specifically address the precision and safety thresholds unique to cerebrovascular interventions while preserving the ergonomic and radiation-mitigating benefits established by earlier cardiovascular platforms.

### Clinical translation and regulatory challenges

Clinical translation of EIRS hinges not only on technical maturity but also on regulatory acceptance and physician adoption. Indeed, clinical studies have demonstrated the safety and feasibility of robotic-assisted interventions in cardiovascular medicine.^253^^,^^254^^,^^255^ However, successful implementation further requires robust human-robot collaboration and retention of clinician authority, together with clearly defined responsibility attribution.^256^^,^^257^ These socio-technical factors collectively determine physician trust and patient acceptance.

With the incorporation of AI and autonomous decision-making into EIRS, these socio-technical requirements become intertwined with emerging ethical and legal challenges. Key concerns include patient autonomy and informed consent, the potential erosion of human surgical judgment due to over-reliance on algorithmic recommendations, algorithmic bias that may exacerbate disparities, and persistent data-privacy and cybersecurity obligations.^258^^,^^259^^,^^260^^,^^261^ Liability attribution becomes harder as autonomy increases, and regulatory frameworks may lag rapid innovation, creating uncertainty around safety standards, approval processes, and post-market surveillance. These risks ultimately materialize within jurisdiction-specific regulatory regimes. Moreover, the high cost of advanced robotic systems raises concerns about equitable access, as their benefits may remain limited to well-resourced healthcare centers.^259^

Against this backdrop, stringent regulatory frameworks across global jurisdictions serve as the gatekeepers of surgical innovation. In the United States, FDA pathways (510(k), *De Novo*, PMA) require substantial equivalence or rigorous safety and efficacy evidence, and are coupled with strictly enforced post-market surveillance. In the European Union, CE marking under the medical device regulation (EU 2017/745) requires conformity assessment, extensive clinical evidence, and post-market clinical follow-up, with time-bound vigilance obligations that vary according to incident severity. Following Brexit, the United Kingdom maintains MHRA oversight under the UKCA marking regime, emphasizing proactive post-market surveillance and imposing stricter incident reporting requirements. Comparable standards apply in Canada, Australia, and New Zealand, each enforcing timely adverse-event reporting and continuous safety monitoring. Japan requires Marketing Authorization Holders to establish dedicated vigilance systems under the Pharmaceuticals and Medical Devices Act, which include prompt reporting of serious adverse events. In China, the National Medical Products Administration enforces rigorous Class II/III registration requirements and mandates timely reporting of deaths and serious injuries through ongoing adverse-event monitoring.

These converging frameworks demonstrate that the principal barrier to EIRS deployment is compliance—regulatory and ethical—rather than technical capability. Many provisions are codified in response to historical patient-safety failures. Accordingly, future EIRS research should not only emphasize technical intelligence and autonomy but also explicitly address clinical responsibility structures, human-in-the-loop collaboration models, and robust compliance strategies aligned with global regulatory norms. Through multidisciplinary collaboration, transparent and interpretable AI, and comprehensive clinician training, EIRS can achieve clinical feasibility and regulatory approval, enabling safe, ethical, and widely accepted adoption in real-world healthcare.

To summarize, advancing the development of fully autonomous EIRS can significantly protect physicians from radiation exposure, reduce their workload, and improve the precision and safety of MIIS. However, to achieve or surpass the performance of experienced human experts, these systems must meet high standards of autonomy. This requires accurate interpretation of patient-specific vascular anatomy and the ability to adapt to dynamic surgical environments. Toward this goal, large AI models offer a promising pathway for enabling intelligent decision-making and real-time adaptation. Nevertheless, their development is currently constrained by limited access to high-quality, diverse datasets. Most current studies rely on simplified, self-constructed vascular phantoms, which are insufficient to validate model robustness and generalization in clinical settings. Therefore, constructing comprehensive datasets that integrate real clinical data with realistic virtual simulations is essential for advancing model training and ensuring clinical relevance.

In parallel, to support the reliable deployment of AI-driven EIRS, standardized evaluation benchmarks must also be established. By incorporating expert-derived clinical criteria for surgical success, autonomous systems can be rigorously evaluated across all relevant performance metrics. This, in turn, will yield robust and credible evidence regarding their clinical efficacy and reliability. Finally, ethical and legal considerations must be addressed proactively, particularly those concerning patient data privacy during data collection and responsibility attribution during autonomous procedures. Developing a robust ethical framework and clear accountability mechanisms will be critical to ensuring the safe, responsible, and effective application of autonomous EIRS into clinical practice.

## Conclusions

As MIIS increasingly becomes the preferred option for the treatment of CCVDs, the limitations of traditional manual methods are becoming more apparent. This shift has driven the development of various types of EIRSs, along with a growing demand for their enhanced autonomy and intelligence. Research into autonomous EIRS has progressed steadily, yielding a series of promising results. This review aims to provide a comprehensive overview of recent progress in the development of autonomous EIRSs. Through a comprehensive analysis of the literature related to endovascular interventions, it can be found that the current research and development focus mainly on master-slave EIRS architectures. These systems are still largely constrained by the procedural logic of traditional manual operations and face several technical challenges. However, this also highlights clear directions for future advancement.

Further research is needed in several key areas, including the development of advanced sensing technologies, the construction of multimodal sensing systems, the development of high-level AI algorithms, and the design of smaller interventional robots featuring strong navigational capabilities and high flexibility. Addressing these areas will be key to achieving higher levels of robotic autonomy in complex clinical environments. In addition, interdisciplinary collaboration is essential to accelerating progress in autonomous EIRS development. Close cooperation among fields such as computer science, mechanical engineering, biomedical engineering, and materials science will provide fresh insights and technical momentum to the evolution of EIRS. Although the development of autonomous EIRS remains in its infancy, existing research has already demonstrated significant potential to overcome the limitations of traditional manual procedures.

With the continuous maturation of related technologies in various fields and a deeper understanding of endovascular interventions, the autonomous operation of EIRS would make significant progress and would be widely applied to a variety of clinical scenarios. Autonomous EIRS is important for improving patient experience, reducing healthcare costs, and promoting healthcare fairness. We believe that the joint efforts of researchers, medical institutions, and enterprises will create a more favorable environment and conditions for the development of autonomous EIRS.

## Acknowledgments

The authors acknowledge the 10.13039/501100001809National Natural Science Foundation of China (62203333 and 52475309), Shanghai Science and Technology Committee (22ZR1465000), Shanghai Municipal Science and Technology Major Project (2021SHZDZX0100), Shanghai Pilot Program for Basic Research, and Fundamental Research Funds for the Shanghai Gaofeng Project for University Academic Program Development.

## Author contributions

J.S. conceived the idea, performed literature search and screening, wrote the original draft of the manuscript, reviewed and edited the manuscript, and assisted with formatting and submission. R.T., Q.Z., and J.L. contributed to literature search and screening, and writing of the original draft. L.G., W.Y., and X.C. provided suggestion and supervision. Y.T. contributed to the conceptualization, supervision, reviewed and edited the manuscript, funding acquisition, and assisted with formatting and submission. All authors read and approved the final version of the manuscript.

## Declaration of interests

The authors declare no competing interests.
